# Purkinje cells in eyeblink conditioning: a systematic review of methodological approaches in animal studies

**DOI:** 10.3389/fnsyn.2026.1957105

**Published:** 2026-10-22

**Authors:** Artem Gornov, Thiago C. Moulin, Anders Rasmussen, Fredrik Johansson

**Affiliations:** 1 Department of Experimental Medical Science, Lund University, Lund, Sweden; 2 Functional Pharmacology and Neuroscience, Department of Surgical Sciences, Uppsala University, Uppsala, Sweden; 3 Department of Pharmaceutical Biosciences, Uppsala University, Uppsala, Sweden

**Keywords:** cerebellum, eye-blink conditioning, methodolgical tools, preclinical research, Purkinje cell, synaptic plasticity

## Abstract

**Background:**

Eyeblink conditioning is a canonical model of associative motor learning and has played a central role in theories of cerebellar plasticity. Purkinje cells occupy a central position in this circuit, receiving conditioned and unconditioned stimulus-related inputs and providing the sole output of the cerebellar cortex. However, the cellular mechanisms by which Purkinje cells contribute to the acquisition, timing, expression, and extinction of conditioned eyeblinks remain debated.

**Methods:**

We systematically reviewed animal studies investigating Purkinje cell mechanisms in eyeblink conditioning to summarize the experimental approaches used in the field, extract qualitative and quantitative evidence, and identify theoretical trends and methodological limitations. A PubMed search identified 184 records, of which 74 met the inclusion criteria. Included studies were classified according to publication features, animal model, conditioning protocol, intervention type, Purkinje cell-related physiological or structural measurements, and proposed learning mechanism.

**Results:**

Most studies either manipulated Purkinje cell function or measured Purkinje cell-associated physiological or structural properties, but only three combined both approaches, revealing a major separation between interventional and physiological evidence. Genetic interventions were predominantly performed in mice, whereas direct Purkinje cell physiological or structural measurement studies were more commonly based on electrophysiological recordings and showed greater model heterogeneity. Most conditioning protocols used parameters compatible with acquisition, although essential details such as interstimulus interval, intertrial interval, or trial number were incompletely reported in a subset of studies. When grouped by publication period, the reviewed studies showed a shift from LTD-centred explanations toward more diverse models that incorporate intrinsic excitability, Purkinje cell pause responses, inhibitory microcircuit mechanisms, cerebellar nuclear plasticity, and olivo-cerebellar feedback.

**Discussion:**

These findings suggest that Purkinje cell contributions to eyeblink conditioning cannot be reduced to a single plasticity mechanism. Instead, the literature supports a multi-level model in which synaptic, intrinsic, and circuit mechanisms jointly shape learning, while also revealing substantial gaps in cross-species validation, reporting quality, and studies combining cell-specific intervention with direct physiological measurement.

## Introduction

Classical eyeblink conditioning is an established experimental paradigm for studying associative learning in the cerebellum. A neutral conditioned stimulus (CS), typically an auditory cue or mild skin stimulation, is repeatedly followed by an unconditioned stimulus (US), such as a corneal air puff or periocular electrical stimulation, which naturally elicits an eyeblink. After repeated pairings, animals begin to produce a conditioned response (CR), consisting of an anticipatory blink response that occurs before the US and is timed to peak near its expected onset. Due to the ease of experimental manipulation of parameters, such as sensory stimuli, the temporal relationship between events, and behavioural output, eyeblink conditioning has been widely used to investigate how learned responses are acquired and expressed by neural circuits (Freeman, 2015).

The cerebellum has long been implicated in eyeblink conditioning. Early lesion and disconnection studies showed that conditioned eyeblink responses depend on cerebellar integrity and helped define the cerebellar structures involved in the acquisition and expression of the CR (Lincoln et al., 1982; McCormick et al., 1985; McCormick and Thompson, 1984a; McCormick and Thompson, 1984b; Norman et al., 1977; Yeo et al., 1985a, 1985b, 1985c). These findings followed theoretical models proposed by Marr and Albus, in which the cerebellar cortex was described as a possible substrate for learning through synaptic plasticity between parallel fibres and Purkinje cells (Albus, 1971; Marr, 1969). Subsequent experiments further clarified the pathways that convey the CS and US to the cerebellum. CS-related information reaches the cerebellar cortex through mossy fibres and granule cell parallel fibres, whereas US-related information is carried by climbing fibres originating in the inferior olive. Replacing the behavioural CS with mossy fibre stimulation and the behavioural US with climbing fibre or inferior olive stimulation can elicit conditioned responses in rabbits and ferrets (Hesslow et al., 1999; Steinmetz et al., 1989; Zucca et al., 2016), supporting the idea that these cerebellar pathways are sufficient to drive associative learning.

Purkinje cells are positioned at the convergence of these two input systems. They receive parallel fibre and climbing fibre inputs and provide the sole output of the cerebellar cortex through inhibitory projections to cerebellar nuclei (Tamanini et al., 2025). This organisation made Purkinje cells natural candidates for mediating learning-related changes in eyeblink conditioning. Changes in their firing can influence the timing and expression of cerebellar nuclear output. Indeed, reported firing patterns are heterogeneous and may contribute to different aspects of the observed behaviour. A well-defined subset of responses consists of a decrease in simple spike firing, a “pause” response that is adaptively timed to the CS-US interval (Jirenhed and Hesslow, 2016). For several decades, the dominant cellular explanation was long-term depression (LTD) at the parallel fibre to Purkinje cell synapse. This model became historically influential as conjunctive activation of parallel fibres and climbing fibres weakens parallel fibre input to Purkinje cells, thereby reducing Purkinje cell inhibition which disinhibits cerebellar nuclei and could facilitate the conditioned response (Hansel et al., 2001; Hesslow et al., 2013; Ito, 2001; Lepora et al., 2010; Medina and Mauk, 2000; Moore et al., 1989). Several studies over the last decades have challenged the sufficiency of this model due to incomplete specificity of molecular manipulations, developmental and compensatory effects in genetic models and the limits of the model in explaining the emergence of precise timing. Synaptic, intrinsic, and circuit-level mechanisms have since been investigated in addition to, or as alternatives to, LTD (Grasselli et al., 2020; Hansel et al., 2001; Hesslow et al., 2013).

The resulting literature contains multiple, partly overlapping hypotheses about how Purkinje cells contribute to conditioned response acquisition, timing, and expression. Thus, the present review systematically examines animal studies investigating Purkinje cell involvement in eyeblink conditioning. We asked what methods are used to investigate the role of Purkinje cells in eyeblink conditioning and which learning mechanisms are experimentally addressed. Such a synthesis allowed us to summarize the information currently available on the subject as well as highlight the unresolved questions and shortcomings of the field.

## Methods

### Search strategy and study selection

We performed a systematic search in PubMed to identify original animal studies investigating the role of Purkinje cells in eyeblink conditioning. The search used the terms “(eyeblink conditioning OR eye-blink conditioning OR nictitating membrane conditioning) AND Purkinje NOT review”, with no publication-year restriction. The initial search was performed on July 6th, 2023, and returned 163 records. During peer review, the search was updated on August 21st, 2026, following the reviewers’ recommendations. Newly identified records were screened using the same eligibility criteria, and eligible studies were incorporated into the data extraction and subsequent analyses. The updated search returned 184 records in total. This systematic review was conducted and reported in accordance with the preferred reporting items for systematic reviews and meta-analyses 2020 statement (PRISMA 2020) (Page et al., 2021).

The first screening step was performed using titles and abstracts. Articles were excluded if they were not written in English or did not present original experimental results, such as reviews or theoretical articles without primary data. Screening was performed independently by two investigators using the Abstrackr platform (Wallace et al., 2012). Articles were retained for full-text screening if at least one reviewer considered them potentially eligible. When the title and abstract did not provide enough information to determine eligibility, the article was retained for full-text assessment.

During full-text screening, studies were included if they investigated Purkinje cell involvement in eyeblink conditioning through at least one of two evidence branches. The first branch, referred to here as the “intervention branch,” included studies in which genetic, pharmacological, lesion-based, or other experimental interventions were used to manipulate Purkinje cell function, Purkinje cell survival, or molecular/circuit mechanisms directly or indirectly related to Purkinje cells. For these studies, the main extracted outcomes were the reported behavioural effects on eyeblink conditioning, including acquisition, retention, extinction, timing, strength, or expression of the conditioned response.

The second branch, referred to here as the Purkinje cell physiological or structural measurement branch, included studies that directly measured Purkinje cell-related variables during or after eyeblink conditioning. This branch, focuses on Purkinje cell readouts, including simple spike activity, complex spike activity, pause responses, intrinsic electrophysiological properties, synaptic measures, and structural/molecular changes. It does not refer to behavioural outcome measures alone.

These two branches were not mutually exclusive. Studies that both manipulated Purkinje cell-related mechanisms and measured Purkinje cell physiological or structural properties were assigned to both branches. Therefore, branch-specific counts may sum to more than the number of unique included studies.

A complete list of screened records, including inclusion status, branch categorization, and reasons for exclusion, is provided in Supplementary Table S1.

### Data extraction

For each included article, we extracted publication features, animal model characteristics, conditioning protocol parameters, intervention features, Purkinje cell readouts, behavioural learning effects, and the learning hypothesis proposed or tested by the authors. Unless otherwise specified, counts refer to unique included studies; analyses based on multiple readouts or protocol entries from the same study are explicitly identified as such.

Publication features included PMID, first author, journal, and publication year. Animal model features included species, strain, sex, and relevant preparations or surgeries. Conditioning protocol features included conditioning type, interstimulus intervals (ISIs), intertrial interval, total number of trials, session structure, omission percentage, and other relevant protocol details when reported.

For studies in the intervention branch, we extracted the type of intervention, the manipulated gene or substance, the control condition, the anatomical or cellular specificity of the intervention, and the reported behavioural effect on eyeblink conditioning. Behavioural effects were classified according to whether learning was impaired, comparable to controls, facilitated, or otherwise altered. Study-level characteristics of intervention studies, including animal model, intervention type and specificity, conditioning paradigm, behavioural effect, and mechanistic classification, are provided in Supplementary Table S2.

For studies in the Purkinje cell measurement branch, we extracted the measurement method, the Purkinje cell variable assessed, the direction of the reported change, and whether the measured variable was related to learning, conditioning, or conditioned response expression. Because some articles measured more than one Purkinje cell variable, readout categories were counted separately when analysing measured Purkinje cell physiological or structural properties. Study-level characteristics of Purkinje cell physiological or structural measurement studies, including animal model, conditioning paradigm, measured Purkinje cell variable, and mechanistic classification, are provided in Supplementary Table S3.

Finally, we extracted the learning mechanism proposed or assumed by each study. This extraction was carried out by a single reviewer and was made only if the text explicitly mentions a hypothesis for the learning mechanism. Papers in which a mechanism was suggested but not explicitly stated were not included in this analysis and were classified as “no mechanism proposed”. Mechanistic hypotheses were then classified as LTD-centred when the study proposed primarily long-term depression at the parallel fibre to Purkinje cell synapse as the main explanatory mechanism. Studies were classified as non-LTD when they proposed, tested, or interpreted learning through mechanisms not restricted to LTD, including bidirectional synaptic plasticity, intrinsic excitability, Purkinje cell pause responses, molecular layer inhibition, cerebellar nuclear plasticity, nucleo-olivary feedback, or distributed/multi-site learning. This binary classification was chosen because LTD represents one of the field’s main hypotheses (Ito, 2001; Lepora et al., 2010; Medina and Mauk, 2000; Moore et al., 1989), whereas the alternatives consist of a highly diverse set of mechanisms that are most practical to analyse as a single group. Articles without a clearly classifiable mechanistic hypothesis were not included in the hypothesis-classification figures. Extracted conditioning protocol parameters, including interstimulus interval, intertrial interval, trial number, omission percentage, and conditioning paradigm, are provided in Supplementary Table S4.

## Results

### Article selection and inclusion

The PubMed search identified 184 records. After title and abstract screening, 50 articles were excluded because they were not original experimental articles or did not involve Purkinje cell intervention or recording. The remaining 134 articles were assessed at the full-text level. Of these, 60 were excluded because they were not original experimental studies, did not involve Purkinje cell intervention or recording, or included Purkinje cell measurements that were not related to learning outcomes. A total of 74 unique studies met the inclusion criteria and were included in the qualitative synthesis (Figure 1).

**Figure 1:**
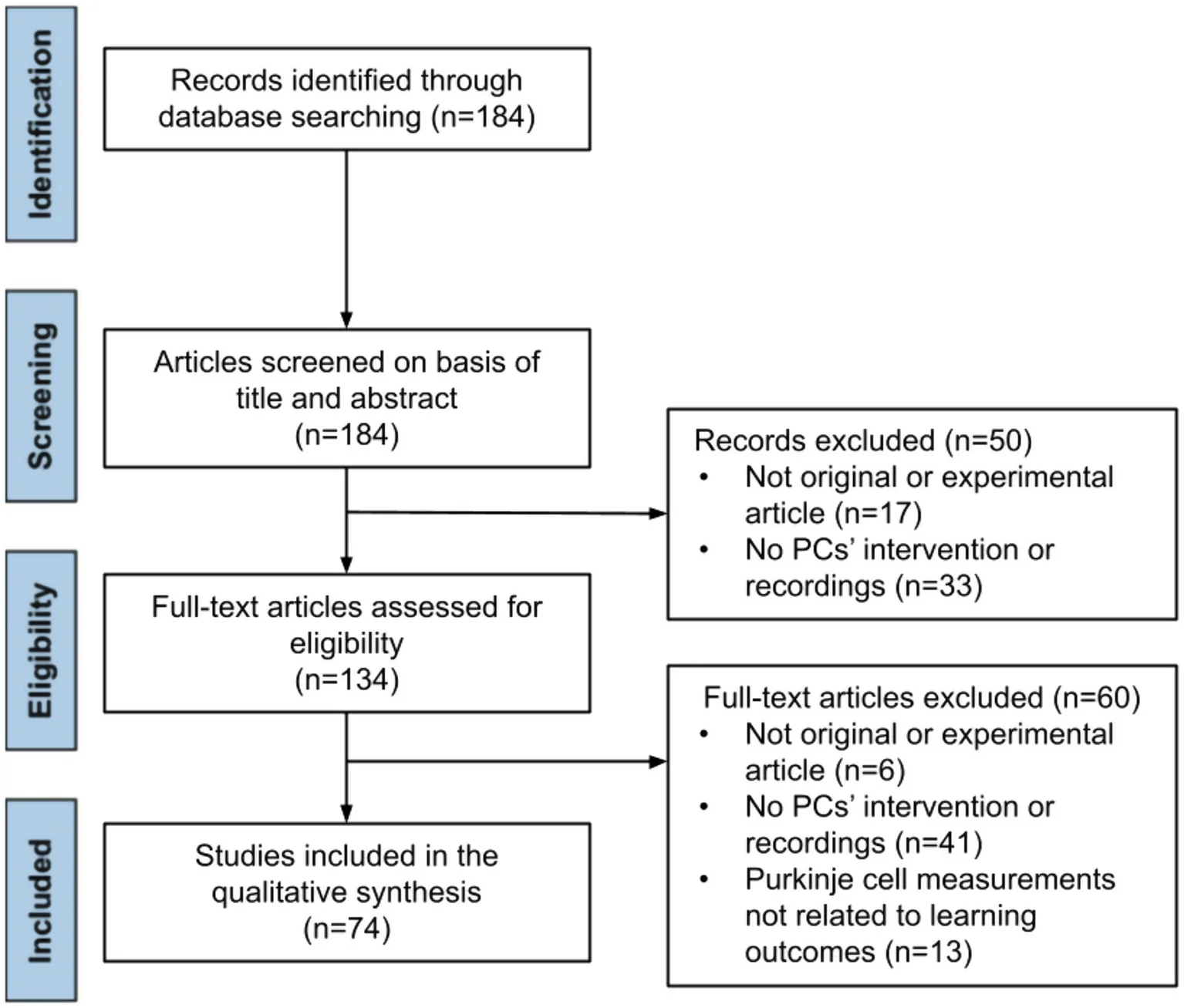
Flowchart of article identification, screening, eligibility assessment, and inclusion. The PubMed search identified 184 records. After title and abstract screening, 50 records were excluded because they were not original experimental articles or did not involve Purkinje cell intervention or recording. The remaining 134 articles were assessed by full-text screening. Of these, 60 were excluded because they were not original experimental articles, did not involve Purkinje cell intervention or recording, or included Purkinje cell measurements unrelated to learning outcomes. A total of 74 unique studies met inclusion criteria and were included in the qualitative synthesis.

### Distribution of studies across intervention and Purkinje cell measurement branches

Among the 74 included studies, 45 (61%) were assigned to the intervention branch and 32 (43%) were assigned to the Purkinje cell physiological or structural measurement branch. These categories were not mutually exclusive: three studies included both an intervention affecting Purkinje cell-related function and direct measurement of Purkinje cell properties. Therefore, branch-specific analyses describe complementary forms of evidence rather than independent groups of studies.

Across publication periods, intervention studies were present throughout the literature and remained stable or increased in the most recent period. In contrast, studies measuring Purkinje cell properties were more concentrated in the 2000s and 2010s, with the highest number of studies published between 2007 and 2016 and fewer studies published since (Figure 2). This suggests that causal/interventional approaches and direct Purkinje cell measurement studies have followed partially different historical trajectories.

**Figure 2:**
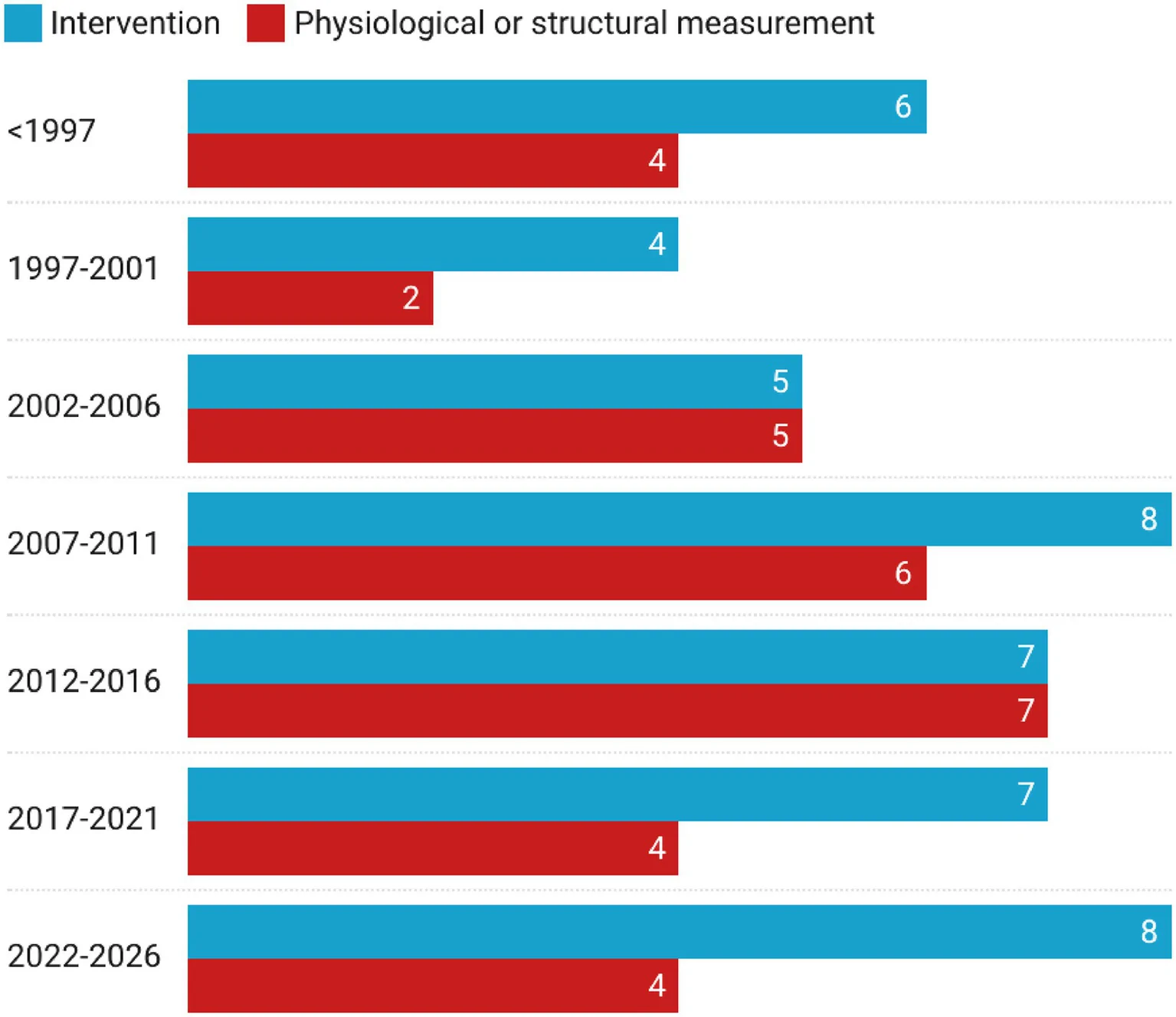
Distribution of intervention-branch and Purkinje cell physiological or structural measurement branch studies across publication periods. Included studies were assigned to one or both evidence branches. The intervention branch includes studies that manipulated Purkinje cell function, survival, or Purkinje cell-related molecular/circuit mechanisms and assessed effects on eyeblink conditioning. The Purkinje cell physiological or structural measurement branch includes studies that directly measured Purkinje cell-related readouts, such as firing activity, pause responses, electrophysiological properties, or structural/molecular changes, during or after eyeblink conditioning. The two branches were not mutually exclusive; studies containing both intervention and direct Purkinje cell measurement contributed to both categories. Bars show the number of studies in each branch for each publication period.

### Conditioning protocols and reporting completeness

Out of the 74 included articles, 66 used delay eyeblink conditioning only, 5 used both delay and trace conditioning and 3 used only trace conditioning. Although trace conditioning differs from delay conditioning in the additional circuitry it recruits (Weiss and Disterhoft, 2011), the small number of trace-conditioning studies did not support meaningful stratified comparisons, and these protocols were therefore not differentiated in further methodological analyses. To evaluate whether the reviewed studies used conditioning protocols compatible with the acquisition of a conditioned eyeblink response, we extracted the main reported protocol parameters, including interstimulus interval, intertrial interval, total number of trials, and omission percentage when available. Most included studies reported sufficient protocol information and used parameters compatible with eyeblink conditioning *in vivo*. However, 10 (13.5%) of the 74 included studies failed to report at least one key protocol parameter, such as the interstimulus interval, intertrial interval, or total number of trials.

Only two articles included protocol conditions with interstimulus intervals below 100 ms, a range in which eyeblink conditioning acquisition is generally considered unreliable (Wetmore et al., 2014). Importantly, both studies tested ISIs across a broader range that also included values at or above 100 ms, rather than relying exclusively on sub-100 ms intervals (Supplementary Figure S1). Thus, the conditioning protocols used in the reviewed literature were broadly compatible with acquisition of conditioned responses. However, incomplete reporting of key parameters, including ISI, ITI, and total trial number, limits reproducibility and prevents more detailed cross-study analyses of how protocol structure may influence learning outcomes.

### Characteristics of intervention-branch studies

The intervention branch was dominated by genetic approaches. Of the 45 intervention studies, 33 (74%) used genetic manipulations, 10 (22%) used pharmacological interventions, 1 study used both and 1 study used a surgical intervention (Figure 3A). All genetic intervention studies were performed in mice, whereas other intervention studies also included rats, ferrets and rabbits. Accordingly, mice were the predominant animal model in the intervention branch, appearing in 35 studies, followed by rats in 6 studies and ferrets and rabbits in 2 studies each (Figure 3B). 15 studies (33%) used males and 17 studies (38%) used animals of both sexes. Over a quarter of the studies (29%, *n* = 13) did not report the sex of the animals used.

**Figure 3:**
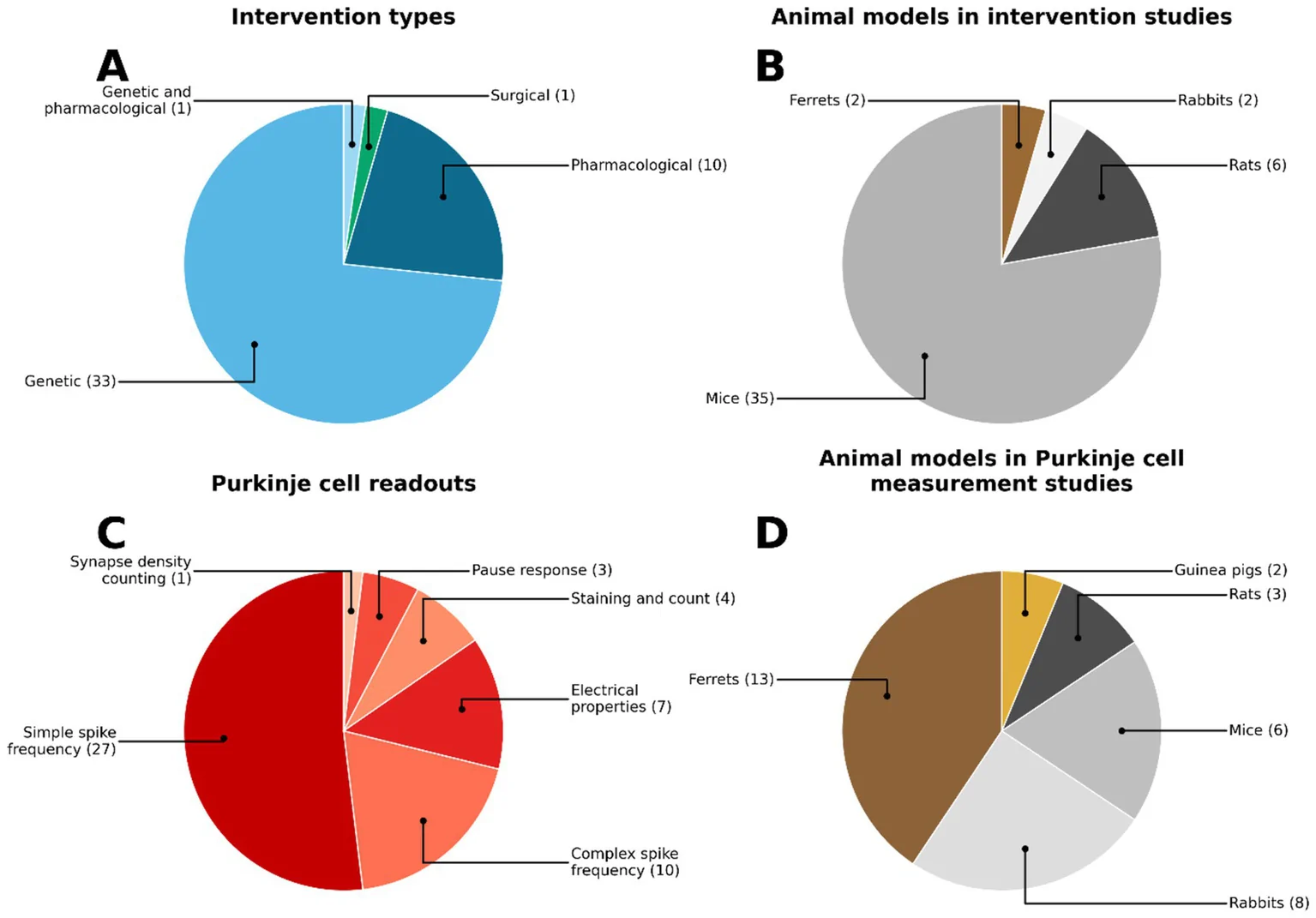
Characteristics of intervention-branch studies and Purkinje cell physiological or structural measurement branch studies. **(A)** Intervention types among studies in the intervention branch. Genetic interventions accounted for most intervention studies, while pharmacological interventions represented a smaller subset. **(B)** Animal models used in the intervention branch studies. Mice were the predominant model, reflecting the dominance of genetic approaches in this branch. **(C)** Purkinje cell readouts extracted from studies in the Purkinje cell physiological or structural measurement branch. Categories include simple spike frequency, complex spike frequency, pause responses, electrical properties, and other Purkinje cell-related measurements. Because some articles measured more than one Purkinje cell variable, this panel represents extracted readout categories rather than mutually exclusive article counts. **(D)** Animal models used in Purkinje cell physiological or structural measurement branch studies. Ferrets were the most common species in studies directly measuring Purkinje cell properties, followed by mice, rabbits, rats, and guinea pigs. Together, these panels show that intervention studies and direct Purkinje cell measurement studies differ both in methodology and in animal-model distribution.

These findings indicate that the causal literature on Purkinje cell involvement in eyeblink conditioning is heavily shaped by mouse genetic models. This provides strong opportunities for molecular and cell-type-specific inference but also creates a species imbalance when compared with the literature directly measuring Purkinje cell physiology.

### Characteristics of Purkinje cell measurement studies

The Purkinje cell measurement branch included 32 studies that directly assessed Purkinje cell-related physiological or structural measurements during or after eyeblink conditioning.

Most studies in this branch used electrophysiological approaches to assess Purkinje cell activity or physiology (88%, n = 28). The extracted Purkinje cell readouts included simple spike frequency, complex spike frequency, pause responses, intrinsic or synaptic electrical properties, and other structural or molecular measures (Figure 3C). Because several articles measured more than one Purkinje cell variable, Figure 3C counts extracted readout categories rather than unique articles. Some articles (28%, *n* = 9) used multiple metrics but never combined different techniques.

Simple spike frequency was the most frequently extracted Purkinje cell readout, followed by electrical properties, complex spike frequency, pause responses, and other measured variables. This indicates that the physiological or structural Purkinje cell measurements branch of the literature is largely a literature of Purkinje cell firing dynamics, especially simple spike modulation, rather than a broad survey of all possible Purkinje cell plasticity mechanisms. The observed responses of Purkinje cells’ simple spike firing rate to CS presentation after conditioning varied between inhibitory, excitatory, non-responsive and biphasic. In all instances where the recorded Purkinje cells were located in HVI and the US was shown to reach the Purkinje cells, the Purkinje cells developed a well-timed pause response.

The animal models used in the Purkinje cell measurement branch differed markedly from those used in the intervention branch. Ferrets were the most common species in this branch, followed by mice, rabbits, rats, and guinea pigs (Figure 3D). 25 studies (78%) used exclusively males, 6 used both males and females (19%), and 1 study did not report the sex of the animals used (3%). Thus, the studies most directly measuring Purkinje cell physiology were not concentrated in the same species as the studies most commonly used for genetic intervention.

### Learning hypotheses across time

Most included studies (82%, *n* = 61) proposed or tested an explicit mechanistic hypothesis for Purkinje cell involvement in eyeblink conditioning. To summarise the evolution of these hypotheses, classifiable studies were grouped into LTD-centred and non-LTD categories. LTD-centred studies were those in which long-term depression at the parallel fibre to Purkinje cell synapse was treated as the primary mechanism of learning, which is considered one of the main hypotheses for the field (Ito, 2001; Lepora et al., 2010; Medina and Mauk, 2000; Moore et al., 1989). Non-LTD studies included those proposing alternative or broader mechanisms, such as bidirectional synaptic plasticity, intrinsic excitability, Purkinje cell pause responses, inhibitory microcircuit mechanisms, cerebellar nuclear plasticity, nucleo-olivary feedback, or distributed learning across multiple cerebellar sites.

In the intervention branch, early studies were dominated by LTD-centred interpretations, whereas more recent studies increasingly included non-LTD or multi-mechanistic explanations (Figure 4A). A similar shift was observed in the Purkinje cell measurement branch, where LTD-centred hypotheses became less common over time and non-LTD explanations dominated the more recent literature (Figure 4B). These results suggest that the field has moved away from a single-mechanism account of Purkinje cell learning and toward a more diverse set of cellular and circuit-level explanations.

**Figure 4:**
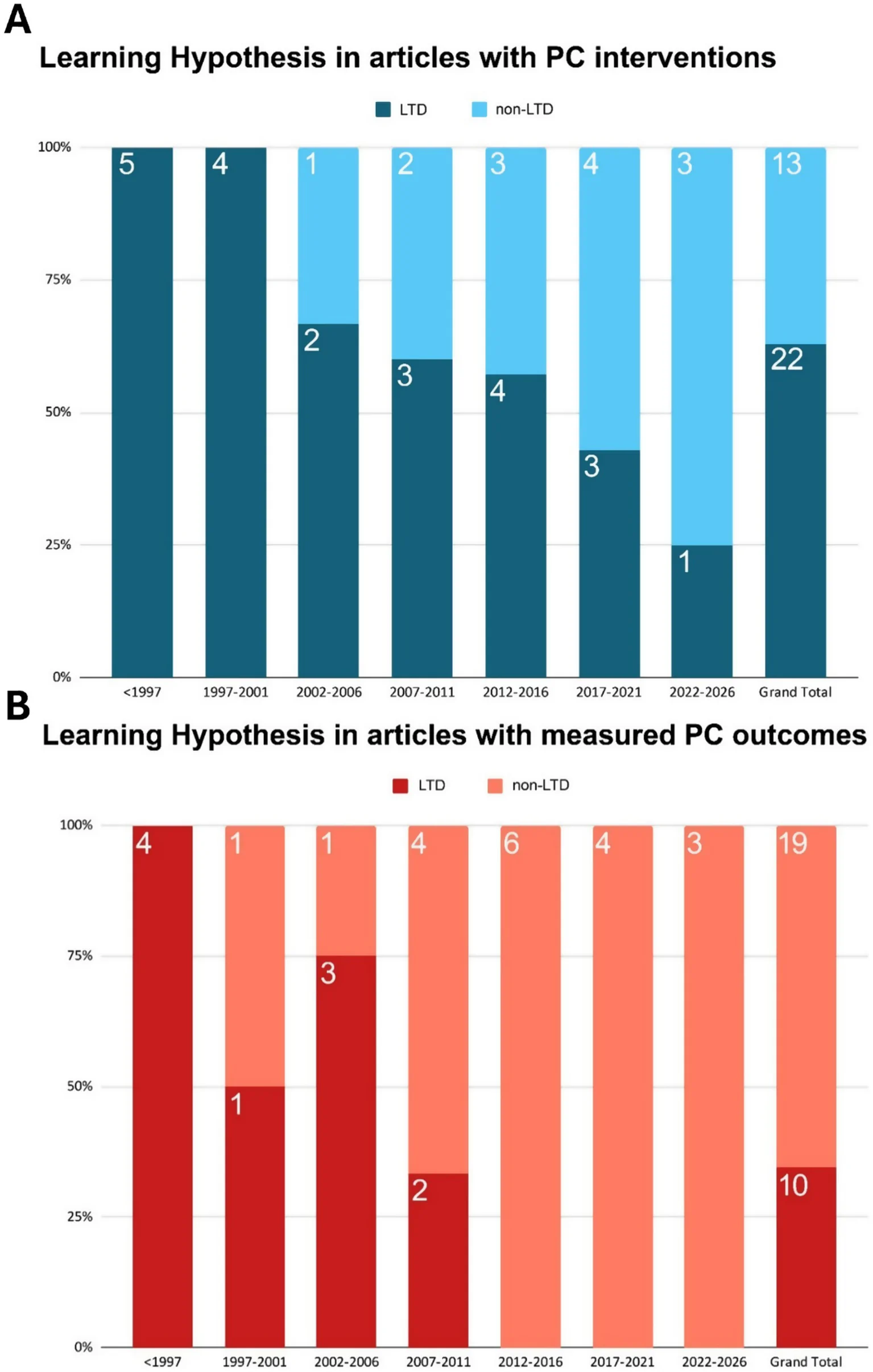
Learning hypotheses in studies with Purkinje cell interventions and measured Purkinje cell physiological or structural properties. **(A)** Distribution of LTD-centred and non-LTD learning hypotheses across publication periods in studies from the intervention branch. **(B)** Distribution of LTD-centred and non-LTD learning hypotheses across publication periods in studies from the Purkinje cell measurement branch. Bars are normalised to 100% within each publication period. Numbers within each segment indicate the absolute number of studies in that category, and the sum of these values gives the total number of classifiable studies contributing to that publication period. The “Total” bar summarises the classifiable studies across all publication periods. LTD-centred studies were those interpreting eyeblink conditioning primarily through long-term depression at the parallel fibre to Purkinje cell synapse. Non-LTD studies included those proposing alternative or broader mechanisms, including bidirectional synaptic plasticity, intrinsic excitability, Purkinje cell pause responses, inhibitory microcircuit mechanisms, cerebellar nuclear plasticity, nucleo-olivary feedback, or distributed/multi-site learning. Articles without a clearly classifiable learning hypothesis were not included in this figure.

## Discussion

In this systematic review, we identified 74 animal studies investigating Purkinje cell involvement in eyeblink conditioning. We organised the literature into two complementary evidence branches, distinguishing studies that intervened on Purkinje cell-related mechanisms from studies that directly measured Purkinje cell physiological or structural properties during or after conditioning. Intervention studies provide evidence that the manipulated molecular, cellular, or circuit-level processes contribute to normal conditioning, whereas measurement studies describe how Purkinje cell activity, physiology, or structure changes in relation to learning. Considered together, these studies support an important role for Purkinje cells in eyeblink conditioning, but they also show that interventional and physiological evidence remain only weakly integrated. Most studies either manipulated Purkinje cell-related function and assessed behavioural learning, or measured Purkinje cell activity and physiology without directly testing causality. Only a small number of studies combined both approaches.

This separation affects how the literature can be interpreted. Genetic, pharmacological, or lesion-based interventions can show that a molecular pathway, cellular process, or Purkinje cell-related circuit component may contribure for normal conditioning. However, when these interventions are not accompanied by direct Purkinje cell recordings or cellular readouts, they do not identify how the manipulation changes Purkinje cell dynamics during learning. Conversely, studies measuring Purkinje cell physiological or structural properties can identify changes in simple spike activity, pause responses, complex spike activity, intrinsic excitability, or structural markers, but they usually cannot determine whether these changes are necessary for learning, compensatory, or only correlated with the conditioned response. Thus, the main limitation of the field is the limited connection between molecular or cellular mechanisms, Purkinje cell dynamics, and behavioural learning within the same experimental framework.

### Search strategy limits

This review does not claim exhaustive coverage of the cerebellar literature. We have limited the search to eyeblink and nictating membrane conditioning and cannot generalize our findings to cerebellar learning as a whole. We also used a single bibliographic database (PubMed) and did not perform citation tracking that could improve the sensitivity of our search.

#### Publication trends and imbalance between evidence branches

The temporal distribution of studies suggests that the field has not expanded steadily, despite the continued absence of consensus on the mechanisms of Purkinje cell learning. Intervention studies were relatively more numerous and remained represented across publication periods, whereas studies directly measuring Purkinje cell properties were more concentrated in earlier periods and appear less frequent in recent years. A likely explanation is the technical difficulty of these experiments. Recording Purkinje cell activity during eyeblink conditioning requires the combination of stable surgical preparations, precise behavioural control, electrophysiological expertise, and analysis of temporally structured neuronal responses. These requirements may explain why the Purkinje cell measurement branch is dominated by electrophysiology and why alternative approaches, such as calcium imaging (which, in its current form, cannot resolve simple-spike pauses), optogenetic perturbation combined with recording, or cell-type-specific molecular readouts, remain comparatively rare in the reviewed literature. As a result, the field has accumulated several candidate mechanisms for Purkinje cell involvement in eyeblink conditioning, but fewer studies have directly examined how these mechanisms are expressed in Purkinje cell activity during learning.

Eyeblink conditioning has long been useful as a model of cerebellar learning because it links a relatively simple behavioural response to a well-defined circuit. The declining representation of direct Purkinje cell measurement studies therefore creates a methodological imbalance. Intervention studies can continue to broaden the list of molecular and circuit mechanisms involved in conditioning, but without a corresponding physiological literature it remains difficult to determine how these mechanisms shape the temporal firing patterns that accompany the learned response.

### Protocol consistency and reporting limitations

The conditioning protocols used in the reviewed studies were generally compatible with acquisition of conditioned eyeblink responses. Studies mostly reported interstimulus intervals, intertrial intervals, and trial numbers within ranges expected to support learning. The main limitation was incomplete and inconsistent reporting of protocol parameters. Several articles, even when studies successfully induced learning, had missing information about ISI, ITI, number of trials, session structure, or acquisition criteria. This is not unique to eyeblink conditioning. Previous systematic reviews of methodological characteristics of behavioural and plasticity-related fields, including fear conditioning (Carneiro et al., 2018) and synaptic plasticity studies (Moulin et al., 2020), have also identified poor or inconsistent reporting of experimental protocols. The present review suggests that eyeblink conditioning studies are affected by a similar problem. In many cases, the available information is sufficient to understand the experiment qualitatively, but not sufficient to support a detailed quantitative comparison across studies. Standardised reporting would improve reproducibility without requiring all laboratories to use identical protocols. Proper reporting of ISI, ITI, trial number, and training schedule is particularly necessary for studies of temporal learning and response timing. Consistent experimental reporting is therefore warranted to improve direct comparison of studies and protocols.

Moreover, although we did not perform a formal risk-of-bias assessment, the reporting limitations identified here provide additional information on the methodological transparency of the literature. A structured evaluation of internal-validity domains such as randomization, blinding, attrition, and sample-size justification would be valuable in future reviews, particularly where quantitative synthesis of study outcomes is intended.

### Animal models and the species-method gap

The intervention branch was dominated by mouse models, probably due to widely available access to genetic tools and their suitability for cell-type-specific and pathway-specific manipulations. Indeed, they have been fundamental for identifying genes, signalling pathways, and Purkinje cell-related mechanisms required for normal eyeblink conditioning. Mouse models are therefore central to the interventional literature on cerebellar learning. However, the same advantage creates a species-method gap (Domeij et al., 2025). The studies most suited for molecular and cell-type-specific inference were not the same studies most often used for direct Purkinje cell physiology. In the reviewed literature, intervention studies were concentrated on mice, whereas Purkinje cell measurement studies were more frequently performed in ferrets and other species. Causal evidence and physiological evidence are therefore separated not only by experimental approach, but also by animal model.

This separation of animal models for distinct experimental approaches complicates syntheses and direct comparisons across the literature. For example, a genetic manipulation in mice may impair conditioning, while electrophysiological studies in ferrets may identify learned changes in Purkinje cell firing. Even when findings are compatible, they do not establish that the manipulated mechanism produces changes similar to those observed in the physiological studies. Similarly, physiological mechanisms identified in larger-animal preparations may not translate directly to mouse genetic models without experimental validation.

Different animal models, therefore, contribute different types of evidence (Domeij et al., 2025). Mice provide genetic access, whereas ferrets, rabbits, and other preparations have historically supported stable recordings of Purkinje cell activity during conditioning. A more integrated literature would require studies in which cell-type-specific interventions and direct Purkinje cell measurements are performed in the same species and behavioural paradigm. Mouse studies combining genetic manipulation with real-time Purkinje cell recording would be particularly useful for testing whether molecular perturbations alter the firing dynamics associated with conditioned response timing and expression. Conversely, selected intervention studies in non-mouse species would help determine whether mechanisms inferred from mouse genetics generalise across cerebellar systems.

### From LTD-centred learning to multi-mechanistic models

Eyeblink conditioning has historically been strongly influenced by the hypothesis that cerebellar learning is driven by long-term depression at the parallel fibre to Purkinje cell synapse. In this model, conjunctive activation of parallel fibre and climbing fibre inputs weakens parallel fibre synapses onto Purkinje cells. This would reduce Purkinje cell inhibition of cerebellar nuclei and contribute to expression of the conditioned response.

The reviewed literature suggests that this LTD-centred model remains important, but is no longer sufficient as a complete explanation of Purkinje cell involvement in eyeblink conditioning (Hesslow et al., 2013). Across both evidence branches, more recent studies increasingly invoke mechanisms beyond classical parallel fibre to Purkinje cell LTD (Boele et al., 2018). These include bidirectional synaptic plasticity, Purkinje cell intrinsic excitability, learned pauses in simple spike firing, complex spike and climbing fibre signalling, feedforward inhibition by molecular layer interneurons, cerebellar nuclear plasticity, nucleo-olivary feedback, and combinations of these mechanisms.

This shift should not be interpreted as a simple rejection of LTD. Rather, the field appears to have moved from asking whether LTD can explain eyeblink conditioning to asking what role LTD plays within a broader learning system. LTD may contribute to some components of learning, but eyeblink conditioning involves multiple behavioural processes, including acquisition through induction of plasticity and storage of the learned association, adaptive timing, response amplitude, motor expression of the response, extinction, and possibly savings or reacquisition. These processes are unlikely to depend on a single plasticity mechanism at one synapse.

The most consistent interpretation is that Purkinje cell learning in eyeblink conditioning is distributed across multiple levels of the cerebellar circuit. Parallel fibre to Purkinje cell plasticity may be one component, but it likely interacts with intrinsic Purkinje cell properties, inhibitory interneuron dynamics, climbing fibre signalling, and cerebellar nuclear plasticity. A unified model will need to explain not only whether Purkinje cells change during conditioning, but how these changes are temporally organised and translated into conditioned behaviour.

### Methodological perspectives

Several methodological gaps emerged from the review. As previously discussed, few studies combined causal manipulation with direct measurement of Purkinje cell activity. This limits the ability to connect molecular or cellular perturbations to Purkinje cell dynamics and behavioural output. Future studies should test whether specific synaptic, intrinsic, or molecular manipulations alter the Purkinje cell firing patterns normally associated with conditioning, and whether those changes predict behavioural deficits. Behavioural endpoints should also be separated more carefully. Acquisition rate, conditioned response amplitude, response timing, expression, extinction, and retention represent different aspects of conditioning and may depend on partially distinct mechanisms. Studies that report only global impairment of learning are therefore difficult to interpret mechanistically. A clearer mapping between Purkinje cell mechanisms and specific behavioural components would allow stronger comparisons across interventions. Moreover, the small number of studies using trace conditioning emerged as an additional gap in the observed methodological diversity. Given the additional circuitry recruited by trace conditioning, this limits the extent to which differences in Purkinje cell mechanisms between delay and trace paradigms can currently be evaluated.

The field would also benefit from closer integration between electrophysiology and newer approaches. Extracellular recordings have provided much of the direct evidence on Purkinje cell activity, but they mainly describe spiking output. These recordings could be complemented by cell-type-specific perturbation, calcium or voltage imaging, synaptic physiology, molecular readouts, and computational models linking cellular changes to behavioural timing. Such approaches would not replace electrophysiology but would help identify the mechanisms that generate the observed firing patterns. Additionally, an important consideration in such cell-level physiological studies is the nested structure of recordings. Multiple Purkinje cells may be obtained from the same animal and should not automatically be treated as independent biological observations, as doing so can alter the interpretation of the statistical evidence. Although we did not formally assess this issue across the included studies, we noted that several reports did not clearly distinguish cell-level from animal-level sampling, reinforcing the need for future studies to report and account for this structure explicitly.

Finally, many mechanistic claims are supported by relatively small numbers of studies, often from specialised laboratories and species-specific preparations. This is understandable given the technical difficulty of the experiments, but it limits the ability to distinguish between competing models. In small literatures, results originating from the same research group cannot always be treated as fully independent evidence, since shared authorship may also reflect shared protocols, analytical decisions, animal preparations, and theoretical expectations (Moulin and Amaral, 2020). More broadly, centralised scientific communities, in which findings are produced by overlapping groups using similar methods, appear less likely to generate replicable claims than more decentralised communities with greater methodological and authorial independence (Danchev et al., 2019). Excessive standardisation within individual laboratories may therefore improve internal consistency while reducing external validity, allowing local methodological conditions to shape the apparent robustness of an effect (Richter et al., 2009; Voelkl et al., 2020). In the context of Purkinje cell mechanisms in eyeblink conditioning, this means that apparent support for particular models may partly reflect the concentration of evidence within specific laboratories, species, preparations, or analytical traditions. Cross-species validation, direct replication, and studies combining intervention with physiological measurement would therefore substantially improve the evidential structure of the field.

## Conclusion

In summary, the reviewed literature supports a central role for Purkinje cells in eyeblink conditioning but does not support a single-mechanism account of Purkinje cell learning. The historical emphasis on parallel fibre to Purkinje cell LTD has progressively given way to a more complex view in which learned Purkinje cell pauses, intrinsic excitability, inhibitory microcircuit dynamics, cerebellar nuclear interactions, and nucleo-olivary feedback may each contribute to different components of conditioned behaviour. The main limitation of the field is no longer the absence of plausible mechanisms, but the fragmentation of evidence across species, preparations, and methodological traditions. Future work will need to connect molecularly specific manipulations with real-time Purkinje cell dynamics and well-defined behavioural endpoints. Only then will it be possible to determine whether Purkinje cells store the memory trace of eyeblink conditioning, implement its timing, regulate its expression, or coordinate these processes as part of a distributed cerebellar learning system.

## Data Availability

All relevant data is contained within the article: The original contributions presented in the study are included in the article and supplementary materials; further inquiries can be directed to the corresponding authors.
