# Supplementary material for: Purkinje cells in eyeblink conditioning: a systematic review of methodological approaches in animal studies
Source: Front Synaptic Neurosci. 2026 Oct 22;18:1957105. doi: 10.3389/fnsyn.2026.1957105 (PMC13736765; doi:10.3389/fnsyn.2026.1957105)
Supplement: Supplementary file 1 [file Image_1.pdf]

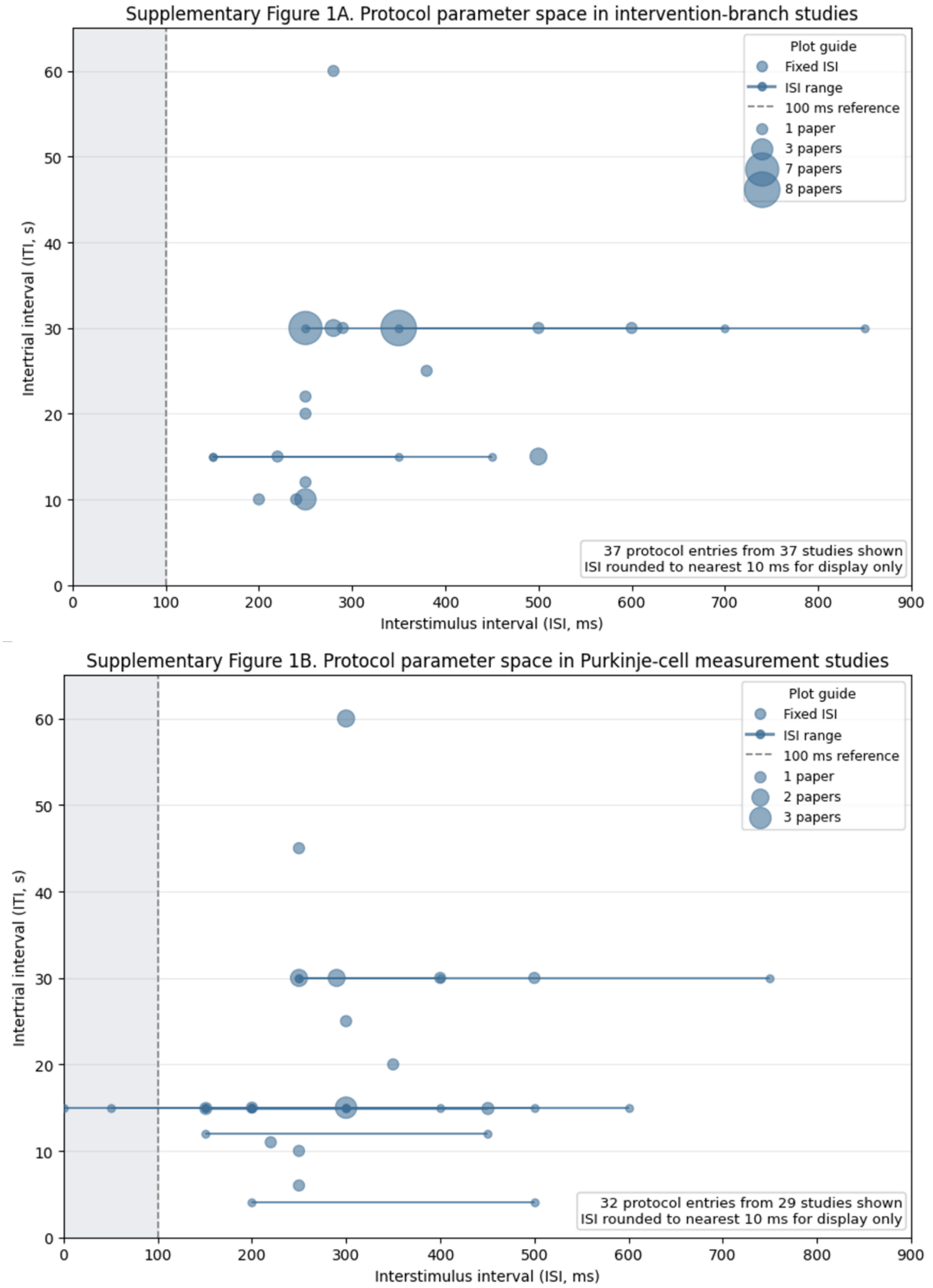

Supplementary Figure 1. Conditioning protocol parameter space among studies reporting both interstimulus interval and intertrial interval. (A) Protocols from intervention-branch studies. **(B)** Protocols from Purkinje cell measurement-branch studies. The x-axis shows the interstimulus interval (ISI, ms), and the y-axis shows the intertrial interval (ITI, s). Circles represent protocol entries with a single fixed ISI. Horizontal line segments represent protocol entries in which a range of ISIs was tested while the ITI was kept constant; the ends of each line indicate the minimum and maximum ISI values. Bubble size indicates the number of studies or protocol entries sharing the same plotted position. For visual clarity, ISI values were rounded to the nearest 10 ms before plotting, while raw extracted values were retained in the dataset. The dashed vertical line marks 100 ms. Two Purkinje cell measurement studies included ISI conditions below this threshold, although both also tested ISIs at or above 100 ms. Only studies or protocol entries reporting both ISI and ITI are shown. Panel A includes 33 protocol entries from 33 intervention-branch studies, and Panel B includes 28 protocol entries from 25 Purkinje cell measurement studies.
