# Supplementary material for: Purkinje cells in eyeblink conditioning: a systematic review of methodological approaches in animal studies
Source: Front Synaptic Neurosci. 2026 Oct 22;18:1957105. doi: 10.3389/fnsyn.2026.1957105 (PMC13736765; doi:10.3389/fnsyn.2026.1957105)
Supplement: Supplementary file 2 [file Table_1.pdf]

PMID	Title	DOI	First Author	Journal/Book	Publication \	Inclusio	Type	Esclusion reason
4043286	Classical conditioning of the nictitating membrane response of the rabbit. II. Lesions of the cerebellar cortex	10.1007/BF00237023	Yeo CH	Exp Brain Res	1985	1	Intervention	
7937862	Learning of the conditioned eye-blink response is impaired by an antisense insulin-like growth factor I oligonucleotide	10.1073/pnas.91.21.10203	Castro-Alamancos MA	Proc Natl Acad Sci U S A	1994	1	Intervention	
7954803	Deficient cerebellar long-term depression and impaired motor learning in mGluR1 mutant mice		Aiba A	Cell	1994	1	Intervention	
8548809	Impaired motor coordination correlates with persistent multiple climbing fiber innervation in PKC gamma mutant mice	10.1016/0092-8674(95)90148-5	Chen C	Cell	1995	1	Intervention	
8785056	Deficient cerebellar long-term depression, impaired eyeblink conditioning, and normal motor coordination in GFAP mutant mice	10.1016/s0896-6273(00)80078-1	Shibuki K	Neuron	1996	1	Intervention	
11285022	Classical eyeblink conditioning in glutamate receptor subunit delta 2 mutant mice is impaired in the delay paradigm but not in the trace paradigm	10.1046/j.0953-816x.2001.01488.x	Kishimoto Y	Eur J Neurosci	2001	1	Intervention	
11403688	Deficient long-term synaptic depression in the rostral cerebellum correlated with impaired motor learning in phospholipase C beta4 mutant mice	10.1046/j.0953-816x.2001.01570.x	Miyata M	Eur J Neurosci	2001	1	Intervention	
11588603	Impaired delay but normal trace eyeblink conditioning in PLCbeta4 mutant mice	10.1097/00001756-200109170-00033	Kishimoto Y	Neuroreport	2001	1	Intervention	
11722613	Impairment of eyeblink conditioning in GluRdelta2-mutant mice depends on the temporal overlap between conditioned and unconditioned stimuli	10.1046/j.0953-816x.2001.01772.x	Kishimoto Y	Eur J Neurosci	2001	1	Intervention	
12106432	The Effect of Kainic Acid Lesions of the Cerebellar Cortex on the Conditioned Nictitating Membrane Response in the Rabbit	10.1111/j.1460-9568.1992.tb00123.x	Hardiman MJ	Eur J Neurosci	1992	1	Intervention	
14500987	Cerebellar LTD and learning-dependent timing of conditioned eyelid responses	10.1126/science.1088383	Koekkoek SK	Science	2003	1	Intervention	
15194823	Cerebellar ataxia and Purkinje cell dysfunction caused by Ca2+-activated K+ channel deficiency	10.1073/pnas.0401702101	Sausbier M	Proc Natl Acad Sci U S A	2004	1	Intervention	
15654300	Vitamin E does not protect against neonatal ethanol-induced cerebellar damage or deficits in eyeblink classical conditioning in rats	10.1097/01.alc.0000150004.53870.e1	Tran TD	Alcohol Clin Exp Res	2005	1	Intervention	
16055059	Deletion of FMR1 in Purkinje cells enhances parallel fiber LTD, enlarges spines, and attenuates cerebellar eyelid conditioning in Fragile X syndrome	10.1016/j.neuron.2005.07.005	Koekkoek SK	Neuron	2005	1	Intervention	
16278298	Normal motor learning during pharmacological prevention of Purkinje cell long-term depression	10.1073/pnas.0508191102	Welsh JP	Proc Natl Acad Sci U S A	2005	1	Intervention	
17923666	Conditioned eyeblink learning is formed and stored without cerebellar granule cell transmission	10.1073/pnas.0708165104	Wada N	Proc Natl Acad Sci U S A	2007	1	Intervention	
17953619	Involvement of protein-tyrosine phosphatase PTPMEG in motor learning and cerebellar long-term depression	10.1111/j.1460-9568.2007.05829.x	Kina S	Eur J Neurosci	2007	1	Intervention	
18256267	Differential regulation of synaptic plasticity and cerebellar motor learning by the C-terminal PDZ-binding motif of GluRdelta2	10.1523/JNEUROSCI.2553-07.2008	Kakegawa W	J Neurosci	2008	1	Intervention	
19931625	Unimpaired trace classical eyeblink conditioning in Purkinje cell degeneration (pcd) mutant mice	10.1016/j.nlm.2009.11.004	Brown KL	Neurobiol Learn Mem	2010	1	Intervention	
20410355	Behavioral characteristics, associative learning capabilities, and dynamic association mapping in an animal model of cerebellar degeneration	10.1152/jn.00180.2010	Porras-García E	J Neurophysiol	2010	1	Intervention	
20797538	Purkinje cell-specific knockout of the protein phosphatase PP2B impairs potentiation and cerebellar motor learning	10.1016/j.neuron.2010.07.009	Schonewille M	Neuron	2010	1	Intervention	
21221776	A new rapid protocol for eyeblink conditioning to assess cerebellar motor learning	10.1007/s11064-010-0392-z	Emi K	Neurochem Res	2011	1	Intervention	
21517144	Impaired eyeblink conditioning in 78 kDa-glucose regulated protein (GRP78)/immunoglobulin binding protein (BiP) conditional knockout mice	10.1037/a0023185	Kim S	Behav Neurosci	2011	1	Intervention	
22988089	Prolonging the postcomplex spike pause speeds eyeblink conditioning	10.1073/pnas.1214274109	Maiz J	Proc Natl Acad Sci U S A	2012	1	Intervention	
24298240	Reevaluation of the role of parallel fiber synapses in delay eyeblink conditioning in mice using Cbln1 as a tool	10.3389/fncir.2013.00180	Emi K	Front Neural Circuits	2013	1	Intervention	
24523559	The synaptic targeting of mGluR1 by its carboxyl-terminal domain is crucial for cerebellar function	10.1523/JNEUROSCI.3542-13.2014	Ohtani Y	J Neurosci	2014	1	Intervention	
25403455	Cerebellar secretin modulates eyeblink classical conditioning	10.1101/lm.035766.114	Fuchs JR	Learn Mem	2014	1	Intervention	
25418414	Cerebellar plasticity and motor learning deficits in a copy-number variation mouse model of autism	10.1038/ncomms6586	Piochon C	Nat Commun	2014	1	Intervention	
26949968	Intracerebellar infusion of the protein kinase M zeta (PKMζ) inhibitor zeta-inhibitory peptide (ZIP) disrupts eyeblink classical conditioning	10.1037/bne0000140	Chihabi K	Behav Neurosci	2016	1	Intervention	
30306129	Impact of parallel fiber to Purkinje cell long-term depression is unmasked in absence of inhibitory input	10.1126/sciadv.aas9426	Boele HJ	Sci Adv	2018	1	Intervention	
31089195	mGluR1 in cerebellar Purkinje cells is essential for the formation but not expression of associative eyeblink memory	10.1038/s41598-019-43744-z	Nakao H	Sci Rep	2019	1	Intervention	
31905212	SK2 channels in cerebellar Purkinje cells contribute to excitability modulation in motor-learning-specific memory traces	10.1371/journal.pbio.3000596	Grasselli G	PLoS Biol	2020	1	Intervention	
32294428	AMPAR Auxiliary Protein SHISA6 Facilitates Purkinje Cell Synaptic Excitability and Procedural Memory Formation	10.1016/j.celrep.2020.03.079	Peter S	Cell Rep	2020	1	Intervention	
33326350	Pavlovian eyeblink conditioning is severely impaired in tottering mice	10.1152/jn.00578.2020	de Oude NL	J Neurophysiol	2021	1	Intervention	
33630615	Intracerebellar infusion of an mGluR1/5 agonist enhances eyeblink conditioning	10.1037/bne0000429	Shipman ML	Behav Neurosci	2021	1	Intervention	
35589396	Dendritic Inhibition by Shh Signaling-Dependent Stellate Cell Pool Is Critical for Motor Learning	10.1523/JNEUROSCI.2073-21.2022	Li W	J Neurosci	2022	1	Intervention	
36352508	Pre-ataxic loss of intrinsic plasticity and motor learning in a mouse model of SCA1	10.1093/brain/awac422	Osório C	Brain	2023	1	Intervention	
37029391	The activation of mGluR4 rescues parallel fiber synaptic transmission and LTP, motor learning and social behavior in a mouse model of Fragile X Syndrome	10.1186/s13229-023-00547-4	Martín R	Mol Autism	2023	1	Intervention	
38165577	Knockdown of the Non-canonical Wnt Gene Prickle2 Leads to Cerebellar Purkinje Cell Abnormalities While Cerebellar-Mediated Behaviors Remain Intact	10.1007/s12311-023-01648-9	Abbott PW	Cerebellum	2024	1	Intervention	
39150780	Mice lacking Astn2 have ASD-like behaviors and altered cerebellar circuit properties	10.1073/pnas.2405901121	Hanzel M	Proc Natl Acad Sci U S A	2024	1	Intervention	
41075059	Deletion of the Voltage-Gated Calcium Channel Gene, Ca(V)1.3, Reduces Purkinje Cell Dendritic Complexity Without Altering Cerebellar-Mediated Eyeblink Conditioning	10.1007/s12311-025-01914-y	Klomp A	Cerebellum	2025	1	Intervention	
42146853	Cerebellar structure and function abnormalities in 16p11.2 microduplication mice	10.1093/braincomms/fcag156	Hayes C	Brain Commun	2026	1	Intervention	
25267641	Memory trace and timing mechanism localized to cerebellar Purkinje cells	10.1073/pnas.1415371111	Johansson F	Proc Natl Acad Sci U S A	2014	1	Intervention + outcome	
32973240	Kir3 channel blockade in the cerebellar cortex suppresses performance of classically conditioned Purkinje cell responses	10.1038/s41598-020-72581-8	Johansson F	Sci Rep	2020	1	Intervention + outcome	
38565684	Climbing fibers provide essential instructive signals for associative learning	10.1038/s41593-024-01594-7	Silva NT	Nat Neurosci	2024	1	Intervention + outcome	
8025262	Suppression of cerebellar Purkinje cells during conditioned responses in ferrets	10.1097/00001756-199401000-00030	Hesslow G	Neuroreport	1994	1	Outcome	
9855491	Purkinje cell responses to pontine stimulation CS during rabbit eyeblink conditioning	10.1016/s0031-9384(98)00217-0	Tracy JA	Physiol Behav	1998	1	Outcome	
10327238	Effects of paired and unpaired eye-blink conditioning on Purkinje cell morphology		Anderson BJ	Learn Mem	1999	1	Outcome	
12692555	Addition of inhibition in the olivocerebellar system and the ontogeny of a motor memory	10.1038/nn1042	Nicholson DA	Nat Neurosci	2003	1	Outcome	
14642645	Purkinje cell activity during learning a new timing in classical eyeblink conditioning	10.1016/j.brainres.2003.09.036	Kotani S	Brain Res	2003	1	Outcome	
14704989	Developmental changes in eyeblink conditioning and simple spike activity in the cerebellar cortex	10.1002/dev.10149	Nicholson DA	Dev Psychobiol	2004	1	Outcome	
15897252	Purkinje cell activity in the cerebellar anterior lobe after rabbit eyeblink conditioning	10.1101/lm.89505	Green JT	Learn Mem	2005	1	Outcome	
16364260	Purkinje cell activity during classical eyeblink conditioning in decerebrate guinea pigs	10.1016/j.brainres.2005.10.090	Kotani S	Brain Res	2006	1	Outcome	
17344387	Acquisition, extinction, and reacquisition of a cerebellar cortical memory trace	10.1523/JNEUROSCI.4202-06.2007	Jirenhed DA	J Neurosci	2007	1	Outcome	
19634946	Eyeblink conditioning leads to fewer synapses in the rabbit cerebellar cortex	10.1037/a0016370	Connor S	Behav Neurosci	2009	1	Outcome	
20032243	Effect of conditioned stimulus parameters on timing of conditioned Purkinje cell responses	10.1152/jn.00524.2009	Svensson P	J Neurophysiol	2010	1	Outcome	
21319893	c-Fos, Arc, and stargazin expression in rat eyeblink conditioning	10.1037/a0022328	Kim S	Behav Neurosci	2011	1	Outcome	
21416378	Learning stimulus intervals--adaptive timing of conditioned purkinje cell responses	10.1007/s12311-011-0264-3	Jirenhed DA	Cerebellum	2011	1	Outcome	
21697357	Time course of classically conditioned Purkinje cell response is determined by initial part of conditioned stimulus	10.1523/JNEUROSCI.1653-11.2011	Jirenhed DA	J Neurosci	2011	1	Outcome	
24478355	Bidirectional plasticity of Purkinje cells matches temporal features of learning	10.1523/JNEUROSCI.2883-13.2014	Wetmore DZ	J Neurosci	2014	1	Outcome	
25140129	Changes in complex spike activity during classical conditioning	10.3389/fncir.2014.00090	Rasmussen A	Front Neural Circuits	2014	1	Outcome	
26504227	Purkinje cell activity during classical conditioning with different conditional stimuli explains central tenet of Rescorla–Wagner model [corrected]	10.1073/pnas.1516986112	Rasmussen A	Proc Natl Acad Sci U S A	2015	1	Outcome	
26551541	Climbing fibers encode a temporal-difference prediction error during cerebellar learning in mice	10.1038/nn.4167	Ohmae S	Nat Neurosci	2015	1	Outcome	
26655909	Evolving Models of Pavlovian Conditioning: Cerebellar Cortical Dynamics in Awake Behaving Mice	10.1016/j.celrep.2015.10.057	ten Brinke MM	Cell Rep	2015	1	Outcome	
26844836	Excitatory Cerebellar Nucleocortical Circuit Provides Internal Amplification during Associative Conditioning	10.1016/j.neuron.2016.01.008	Gao Z	Neuron	2016	1	Outcome	
28533379	Learned response sequences in cerebellar Purkinje cells	10.1073/pnas.1621132114	Jirenhed DA	Proc Natl Acad Sci U S A	2017	1	Outcome	
30283004	Absence of Parallel Fibre to Purkinje Cell LTD During Eyeblink Conditioning	10.1038/s41598-018-32791-7	Johansson F	Sci Rep	2018	1	Outcome	
32015022	Intrinsic Excitability Increase in Cerebellar Purkinje Cells after Delay Eye-Blink Conditioning in Mice	10.1523/JNEUROSCI.2259-19.2019	Titley HK	J Neurosci	2020	1	Outcome	
36405788	Quantitative properties of the creation and activation of a cell-intrinsic duration-encoding engram	10.3389/fncom.2022.1019812	Gallistel CR	Front Comput Neurosci	2022	1	Outcome	
1868333	Learning-specific differences in Purkinje-cell dendrites of lobule HVI (Lobulus simplex): intracellular recording in a rabbit cerebellar slice	10.1016/0006-8993(91)91100-f	Schreurs BG	Brain Res	1991	1	Outcome	
2129873	Purkinje cell number related to rate of classical conditioning	10.1097/00001756-199010000-00020	Woodruff-Pak DS	Neuroreport	1990	1	Outcome	
3758251	Cerebellar Purkinje cell activity related to the classically conditioned nictitating membrane response	10.1007/BF00236851	Berthier NE	Exp Brain Res	1986	1	Outcome	
9120599	Dendritic excitability microzones and occluded long-term depression after classical conditioning of the rabbit's nictitating membrane response	10.1152/jn.1997.77.1.86	Schreurs BG	J Neurophysiol	1997	1	Outcome	
41946969	Neural circuits encode prior knowledge of temporal statistics	10.1038/s41593-026-02255-7	Koppen J	Nat Neurosci	2026	1	Outcome	
10518588	Impaired cerebellar synapse maturation in waggler, a mutant mouse with a disrupted neuronal calcium channel gamma subunit	10.1073/pnas.96.21.12132	Chen L	Proc Natl Acad Sci U S A	1999	0		Intervention/outcome on PCs not linked to EBC
20080589	Differential effects and rates of normal aging in cerebellum and hippocampus	10.1073/pnas.0914207107	Woodruff-Pak DS	Proc Natl Acad Sci U S A	2010	0		Intervention/outcome on PCs not linked to EBC
25205669	Coding of stimulus strength via analog calcium signals in Purkinje cell dendrites of awake mice	10.7554/eLife.03663	Najafi F	Elife	2014	0		Intervention/outcome on PCs not linked to EBC
26063920	Spinocerebellar ataxia type 6 protein aggregates cause deficits in motor learning and cerebellar plasticity	10.1523/JNEUROSCI.0891-15.2015	Mark MD	J Neurosci	2015	0		Intervention/outcome on PCs not linked to EBC
28591570	Compromised Survival of Cerebellar Molecular Layer Interneurons Lacking GDNF Receptors GFRα1 or RET Impairs Normal Cerebellar Motor Learning	10.1016/j.celrep.2017.05.030	Sergaki MC	Cell Rep	2017	0		Intervention/outcome on PCs not linked to EBC
30016703	Cerebellar injury and impaired function in a rabbit model of maternal inflammation induced neonatal brain injury	10.1016/j.nlm.2018.07.005	Zhang Z	Neurobiol Learn Mem	2019	0		Intervention/outcome on PCs not linked to EBC
31582529	The p75NTR Influences Cerebellar Circuit Development and Adult Behavior via Regulation of Cell Cycle Duration of Granule Cell Progenitors	10.1523/JNEUROSCI.0990-19.2019	Zanin JP	J Neurosci	2019	0		Intervention/outcome on PCs not linked to EBC
32985542	Impairment of cerebellar long-term depression and GABAergic transmission in prion protein deficient mice ectopically expressing PrPLP/Dpl	10.1038/s41598-020-72753-6	Kishimoto Y	Sci Rep	2020	0		Intervention/outcome on PCs not linked to EBC
33973524	Differential spatiotemporal development of Purkinje cell populations and cerebellum-dependent sensorimotor behaviors	10.7554/eLife.63668	Beekhof GC	Elife	2021	0		Intervention/outcome on PCs not linked to EBC
37366488	Deficits in cerebellum-dependent learning and cerebellar morphology in male and female BTBR autism model mice	10.3390/neurosci3040045	Kiffmeyer EA	NeuroSci	2022	0		Intervention/outcome on PCs not linked to EBC
8131052	Rabbit cerebellar slice analysis of long-term depression and its role in classical conditioning	10.1016/0006-8993(93)91540-9	Schreurs BG	Brain Res	1993	0		Intervention/outcome on PCs not linked to EBC
16716270	Characteristics of IA currents in adult rabbit cerebellar Purkinje cells	10.1016/j.brainres.2006.04.048	Wang D	Brain Res	2006	0		Intervention/outcome on PCs not linked to EBC
35782305	Cerebellum Involvement in Dystonia During Associative Motor Learning: Insights From a Data-Driven Spiking Network Model	10.3389/fnsys.2022.919761	Geminiani A	Front Syst Neurosci	2022	0		Intervention/outcome on PCs not linked to EBC
8046640	Correspondence between climbing fibre input and motor output in eyeblink-related areas in cat cerebellar cortex	10.1113/jphysiol.1994.sp020126	Hesslow G	J Physiol	1994	0		No intervention/outcome related to Purkinje cells
8046641	Inhibition of classically conditioned eyeblink responses by stimulation of the cerebellar cortex in the decerebrate cat	10.1113/jphysiol.1994.sp020127	Hesslow G	J Physiol	1994	0		No intervention/outcome related to Purkinje cells
8786457	Impaired classical eyeblink conditioning in cerebellar-lesioned and Purkinje cell degeneration (pcd) mutant mice	10.1523/JNEUROSCI.16-08-02829.1996	Chen L	J Neurosci	1996	0		No intervention/outcome related to Purkinje cells
8817254	Inhibition of the inferior olive during conditioned responses in the decerebrate ferret	10.1007/BF00241372	Hesslow G	Exp Brain Res	1996	0		No intervention/outcome related to Purkinje cells
9729243	Selective changes in AMPA receptors in rabbit cerebellum following classical conditioning of the eyelid-nictitating membrane response	10.1016/s0006-8993(98)00525-3	Hauge SA	Brain Res	1998	0		No intervention/outcome related to Purkinje cells
10082638	Essential neuronal pathways for reflex and conditioned response initiation in an intracerebellar stimulation paradigm and the impact of unconditioned stimulus preexposure on learning rate	10.1006/nlme.1998.3872	Swain RA	Neurobiol Learn Mem	1999	0		No intervention/outcome related to Purkinje cells
10197920	Bilateral lesions of the interpositus nucleus completely prevent eyeblink conditioning in Purkinje cell-degeneration mutant mice	10.1037//0735-7044.113.1.204	Chen L	Behav Neurosci	1999	0		No intervention/outcome related to Purkinje cells
11805298	Cerebellar cortical inhibition and classical eyeblink conditioning	10.1073/pnas.032655399	Bao S	Proc Natl Acad Sci U S A	2002	0		No intervention/outcome related to Purkinje cells
11835188	Compartmentation of the rabbit cerebellar cortex	10.1002/cne.10144	Sanchez M	J Comp Neurol	2002	0		No intervention/outcome related to Purkinje cells
12357434	Cerebellar Golgi, Purkinje, and basket cells have reduced gamma-aminobutyric acid immunoreactivity in stargazer mutant mice	10.1002/cne.10406	Richardson CA	J Comp Neurol	2002	0		No intervention/outcome related to Purkinje cells
12492436	mGluR1 in cerebellar Purkinje cells is required for normal association of temporally contiguous stimuli in classical conditioning	10.1046/j.1460-9568.2002.02407.x	Kishimoto Y	Eur J Neurosci	2002	0		No intervention/outcome related to Purkinje cells
12514196	The hippocampus plays an important role in eyeblink conditioning with a short trace interval in glutamate receptor subunit delta 2 mutant mice	10.1523/JNEUROSCI.23-01-00017.2003	Takatsuki K	J Neurosci	2003	0		No intervention/outcome related to Purkinje cells
12582043	Cerebellar mechanisms in eyeblink conditioning	10.1111/j.1749-6632.2002.tb07557.x	Attwell PJ	Ann N Y Acad Sci	2002	0		No intervention/outcome related to Purkinje cells
14573551	GABA neurotransmission in the cerebellar interposed nuclei: involvement in classically conditioned eyeblinks and neuronal activity	10.1152/jn.00859.2003	Aksenov D	J Neurophysiol	2004	0		No intervention/outcome related to Purkinje cells
15506893	Selective developmental increase in the climbing fiber input to the cerebellar interpositus nucleus in rats	10.1037/0735-7044.118.5.1111	Nicholson DA	Behav Neurosci	2004	0		No intervention/outcome related to Purkinje cells
15727524	Purkinje cell loss by OX7-saporin impairs excitatory and inhibitory eyeblink conditioning	10.1037/0735-7044.119.1.190	Nolan BC	Behav Neurosci	2005	0		No intervention/outcome related to Purkinje cells
16719687	Inactivation of sodium channel Scn8A (Na-sub(v)1.6) in Purkinje neurons impairs learning in Morris water maze and delay but not trace eyeblink classical conditioning	10.1037/0735-7044.120.2.229	Woodruff-Pak DS	Behav Neurosci	2006	0		No intervention/outcome related to Purkinje cells
16741286	Purkinje cell loss by OX7-saporin impairs acquisition and extinction of eyeblink conditioning	10.1101/lm.168506	Nolan BC	Learn Mem	2006	0		No intervention/outcome related to Purkinje cells
19218432	Dual involvement of G-substrate in motor learning revealed by gene deletion	10.1073/pnas.0813341106	Endo S	Proc Natl Acad Sci U S A	2009	0		No intervention/outcome related to Purkinje cells
19344639	Genetic manipulation study of information processing in the cerebellum	10.1016/j.neuroscience.2009.01.028	Nakanishi S	Neuroscience	2009	0		No intervention/outcome related to Purkinje cells
19635470	Blocking GABAA neurotransmission in the interposed nuclei: effects on conditioned and unconditioned eyeblinks	10.1016/j.brainres.2009.07.053	Parker KL	Brain Res	2009	0		No intervention/outcome related to Purkinje cells
19747462	Inactivating the middle cerebellar peduncle abolishes the expression of short-latency conditioned eyeblinks	10.1016/j.brainres.2009.08.089	Parker KL	Brain Res	2009	0		No intervention/outcome related to Purkinje cells
21482355	Reevaluating the role of LTD in cerebellar motor learning	10.1016/j.neuron.2011.02.044	Schonewille M	Neuron	2011	0		No intervention/outcome related to Purkinje cells
22198021	Transsynaptic tracing of conditioned eyeblink circuits in the mouse cerebellum	10.1016/j.neuroscience.2011.12.017	Sun LW	Neuroscience	2012	0		No intervention/outcome related to Purkinje cells
22956838	Anatomical characterization of a rabbit cerebellar eyeblink premotor pathway using pseudorabies and identification of a local modulatory network in anterior interpositus	10.1523/JNEUROSCI.2088-12.2012	Gonzalez-Joekes J	J Neurosci	2012	0		No intervention/outcome related to Purkinje cells
23345796	Hippocampal Damage Disrupts Eyeblink Conditioning in Mice Lacking Glutamate Receptor Subunit δ2	10.1023/A:1020362000085	Takatsuki K	J Biol Phys	2002	0		No intervention/outcome related to Purkinje cells
23593266	Age-dependent impairment of eyeblink conditioning in prion protein-deficient mice	10.1371/journal.pone.0060627	Kishimoto Y	PLoS One	2013	0		No intervention/outcome related to Purkinje cells
23680162	Functional inactivation of orexin 1 receptors in the cerebellum disrupts trace eyeblink conditioning and local theta oscillations in guinea pigs	10.1016/j.bbr.2013.05.009	Chen H	Behav Brain Res	2013	0		No intervention/outcome related to Purkinje cells
24706878	Role of granule-cell transmission in memory trace of cerebellum-dependent optokinetic motor learning	10.1073/pnas.1402546111	Wada N	Proc Natl Acad Sci U S A	2014	0		No intervention/outcome related to Purkinje cells
25139623	Cerebellar control of gait and interlimb coordination	10.1007/s00429-014-0870-1	Vinueza Veloz MF	Brain Struct Funct	2015	0		No intervention/outcome related to Purkinje cells
26158416	Cerebellar associative sensory learning defects in five mouse autism models	10.7554/eLife.06085	Kloth AD	Elife	2015	0		No intervention/outcome related to Purkinje cells
26839917	Climbing Fiber Regulation of Spontaneous Purkinje Cell Activity and Cerebellum-Dependent Blink Responses(1,2,3)	10.1523/ENEURO.0067-15.2015	Zucca R	eNeuro	2016	0		No intervention/outcome related to Purkinje cells
27608482	Model-Driven Analysis of Eyeblink Classical Conditioning Reveals the Underlying Structure of Cerebellar Plasticity and Neuronal Activity	10.1109/TNNLS.2016.2598190	Antonietti A	IEEE Trans Neural Netw Learn Syst	2017	0		No intervention/outcome related to Purkinje cells
28512010	Cerebellar learning modulates surface expression of a voltage-gated ion channel in cerebellar cortex	10.1016/j.nlm.2017.05.009	Fuchs JR	Neurobiol Learn Mem	2017	0		No intervention/outcome related to Purkinje cells
29858484	Perineuronal Nets in the Deep Cerebellar Nuclei Regulate GABAergic Transmission and Delay Eyeblink Conditioning	10.1523/JNEUROSCI.3238-17.2018	Hirono M	J Neurosci	2018	0		No intervention/outcome related to Purkinje cells
29934353	Cerebellar Learning Properties Are Modulated by the CRF Receptor	10.1523/JNEUROSCI.3106-15.2018	Ezra-Nevo G	J Neurosci	2018	0		No intervention/outcome related to Purkinje cells
31297821	Complex spike clusters and false-positive rejection in a cerebellar supervised learning rule	10.1113/JP278502	Titley HK	J Physiol	2019	0		No intervention/outcome related to Purkinje cells
31486767	TRPC3 is a major contributor to functional heterogeneity of cerebellar Purkinje cells	10.7554/eLife.45590	Wu B	Elife	2019	0		No intervention/outcome related to Purkinje cells
35805089	mGluR5 Is Substitutable for mGluR1 in Cerebellar Purkinje Cells for Motor Coordination, Developmental Synapse Elimination, and Motor Learning	10.3390/cells11132004	Harbers M	Cells	2022	0		No intervention/outcome related to Purkinje cells
36324646	Sensory Over-responsivity and Aberrant Plasticity in Cerebellar Cortex in a Mouse Model of Syndromic Autism	10.1016/j.bpsgos.2021.09.004	Simmons DH	Biol Psychiatry Glob Open Sci	2021	0		No intervention/outcome related to Purkinje cells
36351971	Cerebellum-dependent associative learning is not impaired in a mouse model of neurofibromatosis type 1	10.1038/s41598-022-21429-4	Ottenhoff MJ	Sci Rep	2022	0		No intervention/outcome related to Purkinje cells
8642419	Metabotropic glutamate receptor activation in cerebellar Purkinje cells as substrate for adaptive timing of the classically conditioned eye-blink response	10.1523/JNEUROSCI.16-11-03760.1996	Fiala JC	J Neurosci	1996	0		No intervention/outcome related to Purkinje cells
8673404	Changes in rabbit cerebellar cortical and interpositus nucleus activity during acquisition, extinction, and backward classical eyelid conditioning	10.1006/nlme.1996.0003	Gould TJ	Neurobiol Learn Mem	1996	0		No intervention/outcome related to Purkinje cells
9037408	Harmaline-induced tremor and impairment of learning are both blocked by dizocilpine in the rabbit	10.1016/s0006-8993(96)01148-1	Du W	Brain Res	1997	0		No intervention/outcome related to Purkinje cells
9438852	Inhibitory cerebello-olivary projections and blocking effect in classical conditioning	10.1126/science.279.5350.570	Kim JJ	Science	1998	0		No intervention/outcome related to Purkinje cells
10798579	Impairment in eyeblink classical conditioning in adult rats exposed to ethanol as neonates		Green JT	Alcohol Clin Exp Res	2000	0		No intervention/outcome related to Purkinje cells
11726241	MRI-assessed volume of cerebellum correlates with associative learning	10.1006/nlme.2001.4026	Woodruff-Pak DS	Neurobiol Learn Mem	2001	0		No intervention/outcome related to Purkinje cells
12445699	Neonatal ethanol produces cerebellar deep nuclear cell loss and correlated disruption of eyeblink conditioning in adult rats	10.1016/s0006-8993(02)03561-8	Green JT	Brain Res	2002	0		No intervention/outcome related to Purkinje cells
12489894	REM sleep deprivation suppresses acquisition of classical eyeblink conditioning		Ohno H	Sleep	2002	0		No intervention/outcome related to Purkinje cells
12867530	Developmental changes in evoked Purkinje cell complex spike responses	10.1152/jn.00481.2003	Nicholson DA	J Neurophysiol	2003	0		No intervention/outcome related to Purkinje cells
14605869	Postnatal expression of Cdkl2 in mouse brain revealed by LacZ inserted into the Cdkl2 locus	10.1007/s00441-003-0828-8	Sassa T	Cell Tissue Res	2004	0		No intervention/outcome related to Purkinje cells
15331619	Glutamate neurotransmission in the cerebellar interposed nuclei: involvement in classically conditioned eyeblinks and neuronal activity	10.1152/jn.00586.2004	Aksenov DP	J Neurophysiol	2005	0		No intervention/outcome related to Purkinje cells
16815479	Stereological estimation of Purkinje neuron number in C57BL/6 mice and its relation to associative learning	10.1016/j.neuroscience.2006.03.070	Woodruff-Pak DS	Neuroscience	2006	0		No intervention/outcome related to Purkinje cells
17671105	Lock-and-key mechanisms of cerebellar memory recall based on rebound currents	10.1152/jn.00344.2007	Wetmore DZ	J Neurophysiol	2008	0		No intervention/outcome related to Purkinje cells
18958191	Lack of Connexin43-mediated bergmann glial gap junctional coupling does not affect cerebellar long-term depression, motor coordination, or eyeblink conditioning	10.3389/neuro.08.001.2008	Tanaka M	Front Behav Neurosci	2008	0		No intervention/outcome related to Purkinje cells
19223439	Secretin effects on cerebellar-dependent motor learning in schizophrenia	10.1176/appi.ajp.2008.08040597	Bolbecker AR	Am J Psychiatry	2009	0		No intervention/outcome related to Purkinje cells
19243062	Motor learning in essential tremor	10.1002/mds.22479	Shill HA	Mov Disord	2009	0		No intervention/outcome related to Purkinje cells
21181461	Dynamic changes in the cerebellar-interpositus/red-nucleus-motoneuron pathway during motor learning	10.1007/s12311-010-0242-1	Sánchez-Campusano R	Cerebellum	2011	0		No intervention/outcome related to Purkinje cells
23398437	Cerebellar structure and function in male Wistar-Kyoto hyperactive rats	10.1037/a0031897	Thanellou A	Behav Neurosci	2013	0		No intervention/outcome related to Purkinje cells
23764510	Aging in the cerebellum and hippocampus and associated behaviors over the adult life span of CB6F1 mice	10.1016/j.neuroscience.2013.06.002	Kennard JA	Neuroscience	2013	0		No intervention/outcome related to Purkinje cells
23982588	Golgi cell activity during eyeblink conditioning in decerebrate ferrets	10.1007/s12311-013-0518-3	Rasmussen A	Cerebellum	2014	0		No intervention/outcome related to Purkinje cells
24931828	Localization of the cerebellar cortical zone mediating acquisition of eyeblink conditioning in rats	10.1016/j.nlm.2014.06.003	Steinmetz AB	Neurobiol Learn Mem	2014	0		No intervention/outcome related to Purkinje cells
25390365	Adaptive robotic control driven by a versatile spiking cerebellar network	10.1371/journal.pone.0112265	Casellato C	PLoS One	2014	0		No intervention/outcome related to Purkinje cells
25973604	Cerebellar-dependent associative learning is preserved in Duchenne muscular dystrophy: a study using delay eyeblink conditioning	10.1371/journal.pone.0126528	Schara U	PLoS One	2015	0		No intervention/outcome related to Purkinje cells
28319608	Cerebellar granule cells acquire a widespread predictive feedback signal during motor learning	10.1038/nn.4531	Giovannucci A	Nat Neurosci	2017	0		No intervention/outcome related to Purkinje cells
28858616	Inferior Olivary TMEM16B Mediates Cerebellar Motor Learning	10.1016/j.neuron.2017.08.010	Zhang Y	Neuron	2017	0		No intervention/outcome related to Purkinje cells
29243588	Dynamic modulation of activity in cerebellar nuclei neurons during pavlovian eyeblink conditioning in mice	10.7554/eLife.28132	Ten Brinke MM	Elife	2017	0		No intervention/outcome related to Purkinje cells
29680227	Cerebellar transcranial direct current stimulation interacts with BDNF Val66Met in motor learning	10.1016/j.brs.2018.04.009	van der Vliet R	Brain Stimul	2018	0		No intervention/outcome related to Purkinje cells
31560921	Intracerebellar cannabinoid administration impairs delay but not trace eyeblink conditioning	10.1016/j.bbr.2019.112258	Steinmetz AB	Behav Brain Res	2020	0		No intervention/outcome related to Purkinje cells
33247191	A FN-MdV pathway and its role in cerebellar multimodular control of sensorimotor behavior	10.1038/s41467-020-19960-x	Wang X	Nat Commun	2020	0		No intervention/outcome related to Purkinje cells
34582972	Non-invasive recording from the human cerebellum during a classical conditioning paradigm using the otolith-evoked blink reflex	10.1016/j.neulet.2021.136270	Todd NPM	Neurosci Lett	2021	0		No intervention/outcome related to Purkinje cells
34790271	Influence of various temporal recoding on pavlovian eyeblink conditioning in the cerebellum	10.1007/s11571-021-09673-2	Kim SY	Cogn Neurodyn	2021	0		No intervention/outcome related to Purkinje cells
35129170	A Flexible Platform for Monitoring Cerebellum-Dependent Sensory Associative Learning	10.3791/63205	Broussard GJ	J Vis Exp	2022	0		No intervention/outcome related to Purkinje cells
40603888	Processing reliant on granule cells is essential for motor learning but dispensable for social preference and numerous other cerebellar-dependent behaviors	10.1038/s41467-025-61190-6	Lee JH	Nat Commun	2025	0		No intervention/outcome related to Purkinje cells
10456059	A model of Pavlovian eyelid conditioning based on the synaptic organization of the cerebellum	10.1101/lm.4.1.130	Mauk MD	Learn Mem	1997	0		Not original / model
11907559	Neurobiology: ready to unlearn	10.1038/416270a	Kitazawa S	Nature	2002	0		Not original / model
12645844	Subcellular interactions between parallel fibre and climbing fibre signals in Purkinje cells predict sensitivity of classical conditioning to interstimulus interval	10.1007/BF02734249	Kotaleski JH	Integr Physiol Behav Sci	2002	0		Not original / model
14500971	Neuroscience. From molecules to memory in the cerebellum	10.1126/science.1090462	Linden DJ	Science	2003	0		Not original / model
15073358	Comment on "Cerebellar LTD and learning-dependent timing of conditioned eyelid responses"	10.1126/science.304.5668.211a	Christian KM	Science	2004	0		Not original / model
15306737	A biophysical model of synaptic delay learning and temporal pattern recognition in a cerebellar Purkinje cell	10.1023/B:JCNS.0000037678.26155.b5	Steuber V	J Comput Neurosci	2004	0		Not original / model
18648667	Interaction between Purkinje cells and inhibitory interneurons may create adjustable output waveforms to generate timed cerebellar output	10.1371/journal.pone.0002770	Hong S	PLoS One	2008	0		Not original / model
21614100	Neural circuit and its functional roles in cerebellar cortex	10.1007/s12264-011-1044-2	Wang L	Neurosci Bull	2011	0		Not original / model
22438912	A computational mechanism for unified gain and timing control in the cerebellum	10.1371/journal.pone.0033319	Yamazaki T	PLoS One	2012	0		Not original / model
24590391	Modeling possible effects of atypical cerebellar processing on eyeblink conditioning in autism	10.3758/s13415-014-0263-1	Radell ML	Cogn Affect Behav Neurosci	2014	0		Not original / model
26479712	Theoretical considerations for understanding a Purkinje cell timing mechanism	10.4161/19420889.2014.994376	Johansson F	Commun Integr Biol	2014	0		Not original / model
26949723	Mechanisms for motor timing in the cerebellar cortex	10.1016/j.cobeha.2016.01.013	Johansson F	Curr Opin Behav Sci	2016	0		Not original / model
28264639	A Multiple-Plasticity Spiking Neural Network Embedded in a Closed-Loop Control System to Model Cerebellar Pathologies	10.1142/S0129065717500174	Geminiani A	Int J Neural Syst	2018	0		Not original / model
29391392	A cerebellar mechanism for learning prior distributions of time intervals	10.1038/s41467-017-02516-x	Narain D	Nat Commun	2018	0		Not original / model
32040505	A model for time interval learning in the Purkinje cell	10.1371/journal.pcbi.1007601	Majoral D	PLoS Comput Biol	2020	0		Not original / model
33961660	A biochemical mechanism for time-encoding memory formation within individual synapses of Purkinje cells	10.1371/journal.pone.0251172	Mandwal A	PLoS One	2021	0		Not original / model
34975423	Cortico-Cerebellar Hyper-Connections and Reduced Purkinje Cells Behind Abnormal Eyeblink Conditioning in a Computational Model of Autism Spectrum Disorder	10.3389/fnsys.2021.666649	Trimarco E	Front Syst Neurosci	2021	0		Not original / model
36950506	A computational passage-of-time model of the cerebellar Purkinje cell in eyeblink conditioning	10.3389/fncom.2023.1108346	Ricci M	Front Comput Neurosci	2023	0		Not original / model
38405978	Mice lacking Astn2 have ASD-like behaviors and altered cerebellar circuit properties	10.1101/2024.02.18.580354	Hanzel M	bioRxiv	2024	0		Not original / model
38574161	Mesoscale simulations predict the role of synergistic cerebellar plasticity during classical eyeblink conditioning	10.1371/journal.pcbi.1011277	Geminiani A	PLoS Comput Biol	2024	0		Not original / model
40196480	Deletion of the voltage-gated calcium channel gene, Ca (V) 1.3, reduces Purkinje cell dendritic complexity without altering cerebellar-mediated eyeblink conditioning	10.1101/2025.03.27.645586	Klomp AJ	bioRxiv	2025	0		Not original / model
40520650	Modeling nitric oxide diffusion and plasticity modulation in cerebellar learning	10.1063/5.0250953	Trapani AM	APL Bioeng	2025	0		Not original / model
41036515	A theory of cerebellar learning as spike-based reinforcement learning in continuous time and space	10.1093/pnasnexus/pgaf302	Kuriyama R	PNAS Nexus	2025	0		Not original / model

Supplementary table S1. Screened records
