# Supplementary material for: Purkinje cells in eyeblink conditioning: a systematic review of methodological approaches in animal studies
Source: Front Synaptic Neurosci. 2026 Oct 22;18:1957105. doi: 10.3389/fnsyn.2026.1957105 (PMC13736765; doi:10.3389/fnsyn.2026.1957105)
Supplement: Supplementary file 3 [file Table_2.pdf]

**Supplementary table 2.** Study-level characteristics of intervention studies

| PMID | Species | Sex | Strain | Intervention type | Specificity | Conditioning paradigm | Effect on conditioning | Mechanistic classification |
|---|---|---|---|---|---|---|---|---|
| 25267641 | ferrets | Male | ND | Pharmacological | Cerebellum | Delay | Comparable | not LTD, intracellular learning |
| 32973240 | ferrets | Male | ND | Pharmacological | Cerebellum | Delay | Impaired | Intracellular learning |
| 39150780 | mice | Both | Astn2 KO | Genetic | Both PC and whole brain | Delay | Impaired | - |
| 17923666 | mice | ND | TeNT / Tet mice | Genetic | Cerebellum | Delay | Comparable | Interpositus nucleus learning |
| 20410355 | mice | Male | Lucher mutant (+/Lc) | Genetic | Cerebellum | Delay | Comparable | - |
| 24298240 | mice | Male | cbln1-/- | Genetic | Cerebellum | Delay | Impaired | LTD |
| 14500987 | mice | ND | L7-PKC +/- | Genetic | PCs | Delay | Impaired | LTD |
| 16055059 | mice | ND | L7-Fmr1 mutant | Genetic | PCs | Delay | Impaired | LTD |
| 19931625 | mice | Male | B6.BRAgtpbp1pcd/J | Genetic | PCs | Trace | Comparable | - |
| 20797538 | mice | ND | PP2B-loxP/L7-Cre | Genetic | PCs | Delay | Impaired | LTP, Intrinsic excitability |
| 21517144 | mice | Male | Grp78 F/F, F/- | Genetic | PCs | Delay | Impaired | - |
| 24523559 | mice | Both | mGluR1-KO / L7-mGluR1b | Genetic | PCs | Delay | Impaired | LTD |
| 30306129 | mice | Both | GluR2Δ7-L7-Δγ2 | Genetic | PCs | Delay | Impaired | LTD and FFI combination |
| 31089195 | mice | Both | mGluR1−/−/L7-tTA/TRE-mGluR1a: mGluR1 cKO | Genetic | PCs | Delay | Impaired | LTD |
| 31905212 | mice | Both | L7-SK2 KO | Genetic | PCs | Delay | Impaired | Intrinsic excitability |
| 32294428 | mice | Both | L7(Pcp2)-Cre x Shisa6-tm1a2(Caliper)CNCR-VUA | Genetic | PCs | Delay | Impaired | LTP |
| 36352508 | mice | Both | ATXN1[82Q] (Tg(Pcp2-ATXN1*82Q) 5Horr) | Genetic | PCs | Delay | Impaired | Intrinsic excitability |
| 33326350 | mice | Both | tottering (tg) | Genetic | Systemic | Delay | Impaired | Intrinsic excitability |
| 7954803 | mice | ND | mGluR1-KO | Genetic | Whole brain | Delay | Impaired | LTD |
| 8548809 | mice | ND | PKCgamma mutant | Genetic | Whole brain | Delay | Facilitated | LTD, CF innervation |
| 8785056 | mice | Male | GFAP -/- | Genetic | Whole brain | Delay | Impaired | LTD |
| 11285022 | mice | ND | GluRd2-/- | Genetic | Whole brain | Delay and Trace | Impaired | LTD |
| 11403688 | mice | ND | PLCβ4-/- | Genetic | Whole brain | Delay | Impaired | LTD |
| 11588603 | mice | Both | PLCβ4-/- | Genetic | Whole brain | Delay and Trace | Impaired | LTD |
| 11722613 | mice | ND | GluRd2-/- | Genetic | Whole brain | Delay and Trace | Impaired | LTD |
| 15194823 | mice | Both | BK-/- | Genetic | Whole brain | Delay | Impaired | - |
| 17953619 | mice | ND | PTPMEG-KO | Genetic | Whole brain | Delay | Impaired | LTD |
| 18256267 | mice | Male | GluRd2-/- /TgΔCT7 | Genetic | Whole brain | Delay | Impaired | LTD |
| 21221776 | mice | ND | hotfoot-5J | Genetic | Whole brain | Delay | Impaired | LTD |
| 25418414 | mice | Both | patDp/+ | Genetic | Whole brain | Delay | Impaired | LTD, not LTP |
| 35589396 | mice | Both | Ptf1aCre/+; Smo F/- | Genetic | Whole brain | Delay | Impaired | FFI |
| 37029391 | mice | Male | Fmr1 KO | Genetic | Whole brain | Delay | Impaired | LTP |
| 38165577 | mice | Both | Prickle2-/- | Genetic | Whole brain | Delay | Comparable | - |
| 41075059 | mice | Both | CaV1.3 -/- | Genetic | Whole brain | Delay | Comparable | - |
| 42146853 | mice | Both | 16p11.2dp /+ | Genetic | Whole brain | Delay | Impaired | - |
| 38565684 | mice | Both | WT, Chr2-PC, Chr2-Gabra6, Chr2-vGlut2 | Genetic and pharmacological | Regional cerebellar cortex | Delay | Impaired | LTD |
| 12106432 | rabbit | Male | Dutch-Belted WT | Pharmacological | Regional cerebellar cortex | Delay | Impaired | - |
| 4043286 | rabbit | Male | Dutch-Belted WT | Surgical | Regional cerebellar cortex | Delay | Impaired | LTD |
| 7937862 | rats | Male | Wistar | Pharmacological | Cerebellum | Delay | Impaired | LTD |
| 22988089 | rats | ND | ND | Pharmacological | Cerebellum | Delay | Facilitated | Pause response, Intrinsic excitability |
| 25403455 | rats | Male | Wistar | Pharmacological | Cerebellum | Delay | Impaired | FFI |
| 26949968 | rats | Male | Wistar | Pharmacological | Cerebellum | Delay | Impaired | LTP |
| 33630615 | rats | Male | Wistar | Pharmacological | Cerebellum | Delay | Facilitated | LTD |
| 15654300 | rats | Both | Long-Evans | Pharmacological | Whole brain | Delay | Impaired | - |
| 16278298 | rats | ND | Sprague-Dawley albino | Pharmacological | Whole brain | Delay | Comparable | not LTD, CF innervation |
