# Supplementary material for: Purkinje cells in eyeblink conditioning: a systematic review of methodological approaches in animal studies
Source: Front Synaptic Neurosci. 2026 Oct 22;18:1957105. doi: 10.3389/fnsyn.2026.1957105 (PMC13736765; doi:10.3389/fnsyn.2026.1957105)
Supplement: Supplementary file 4 [file Table_3.pdf]

Supplementary table 3. Study-level characteristics of Purkinje cell physiological or structural measurement studies

PMID	Species	Strain	Sex	Conditioning paradigm	Outcome type	Mechanistic classification
25140129	ferret	WT	male	Delay	Cpx Hz	NO feedback
26504227	ferret	WT	male	Delay	Simple Hz	Interaction IO-PC
21697357	ferret	WT	male	Delay	Simple Hz	Intracellular learning
24478355	ferret	WT	male	Delay	Simple Hz	Intracellular learning
30283004	ferret	WT	male	Delay	Simple Hz	Not LTD, intracellular learning
21416378	ferret	WT	male	Delay	Simple Hz	Not delay line model (Not LTD)
17344387	ferret	WT	male	Delay	Simple Hz	Intracellular learning
8025262	ferret	WT	male	Delay	Simple Hz	LTD
32973240	ferret	WT	male	Delay	Simple Hz	Intracellular learning mGlur route
20032243	ferret	WT	male	Delay	Simple Hz	Cortical interneurons
28533379	ferret	WT	male	Delay	Simple Hz	Intracellular learning
25267641	ferret	WT	male	Delay	Simple Hz and Pause	Intracellular learning
36405788	ferret	WT	male	Delay and Trace	Simple Hz and Pause	Intracellular learning
14642645	guinea pig	WT	male	Delay	Simple Hz	-
16364260	guinea pig	WT	male	Delay	Simple Hz	LTD
26551541	mouse	WT	male	Delay	Cpx Hz	CF encoding
26844836	mouse	WT	both	Delay	PC electrical propoerties	Circuitry level feedback/feedforward learning
32015022	mouse	WT	male	Delay	PC electrical propoerties	Intrinsic Plasticity
26655909	mouse	WT	male	Delay	Simple Hz and Cpx Hz	Multifactorial learning with MLI and NO involvment
38565684	mouse	WT (C57BL/6J) + Cre transgenics (L7-Cre;ChR2, Gabra6-Cre;ChR2, vGlut2-Cre;ChR2)	both	Delay	Simple Hz/Cpx Hz	CF encoding
41946969	mouse	WT (C57BL/6) + L7-Cre;Ai32 (PCP2-Cre-ChR2-eYFP)	both	Trace	Simple Hz/Cpx Hz and other	LTD and LTP at GC-Purkinje-cell synapses (computational model for encoding temporal priors)
1868333	rabbit	albino	male	Delay	Dendrite excitability	LTD
19634946	rabbit	albino	male	Delay	other	LTD
10327238	rabbit	WT	male	Delay	PC count	PF-PC and CF-PC plasticity
2129873	rabbit	WT	both	Trace	PC count	-
9120599	rabbit	albino	male	Delay	PC electrical propoerties	Membrane and synaptic excitability
15897252	rabbit	albino	male	Delay	Simple Hz	LTP and LTD
9855491	rabbit	WT	male	Delay then Trace	Simple Hz	LTD
3758251	rabbit	albino	ND	Delay	Simple Hz/Cpx Hz	LTD
12692555	rat	WT	both	Delay	Cpx Hz	CF encoding
21319893	rat	WT	male	Delay	PC count	LTD
14704989	rat	WT	both	Delay	Simple Hz	LTD and LTP
