# Supplementary material for: Purkinje cells in eyeblink conditioning: a systematic review of methodological approaches in animal studies
Source: Front Synaptic Neurosci. 2026 Oct 22;18:1957105. doi: 10.3389/fnsyn.2026.1957105 (PMC13736765; doi:10.3389/fnsyn.2026.1957105)
Supplement: Supplementary file 5 [file Table_4.pdf]

Supplementary Table S4. Conditioning protocol parameters

PMID	ISI	ITI	Trials	Omissions	Protocol
1868333	300	60	240	0	Delay
2129873	500	30	until learning	0	Trace
3758251	350	20	until learning	0	Delay
4043286	500	15	1000	0	Delay
7937862	380	25	100000	0.1	Delay
7954803	280	60	500	0.1	Delay
8025262	300	25	until leraning	0	Delay
8548809	252	30	700	0.2	Delay
8785056	250	30	800	0.2	Delay
9120599	300	60	240	0	Delay
9855491	250	NOT REPORTED	until learning	0.1	Delay
10327238	250	30	>1000	0.11	Delay
11285022	252-852	30	700	0.1	Delay and Trace
11403688	250	NOT REPORTED	600	0.1	Delay
11588603	252	NOT REPORTED	700	0.1	Delay and Trace
11722613	352	30	until learning	0.1	Delay and Trace
12106432	500	15	1000	0.1	Delay
12692555	294	30	500	0.2	Delay
14500987	350	30	400	0.2	Delay
14642645	250-400	30	>1200	0.1	Delay
14704989	294	30	500	0.2	Delay
15194823	NOT REPORTED	NOT REPORTED	500	0.1	Delay
15654300	280	30	600	0.1	Delay
15897252	250-750	30	1920+1080	0.4	Delay then Trace
16055059	500	30	256	0.25	Delay
16278298	350-700	30	616	0.1	Delay
16364260	400	30	480	0.1	Delay
17344387	200-300	15	40-900	0	Delay
17923666	352	30	700	0.1	Delay
17953619	350	30	700	0.1	Delay
18256267	250	30	700	0.1	Delay
19634946	250	NOT REPORTED	until learning	0.2	Delay
19931625	250	22	1000	0.1	Delay
20032243	200-500	15	until learning	0	Delay
20032243	300	15	until learning	0	Delay
20410355	250	30	540-900	0.1	Delay
20797538	350	30	256	0.25	Delay
21221776	250	30	600	0.1	Delay
21319893	252	30	100	0.2	Delay
21416378	200-600	15	200-800	0	Delay
21517144	252	30	1000	0.2	Delay
21697357	200-400	15	until learning	0	Delay
22988089	290	30	600	0.1	Delay
24298240	250	30	700	0.1	Delay
24478355	0-300	15	variable	0	Delay
24523559	350	30	700	0.1	Delay
25140129	50-150	15	240	0	Delay
25140129	300	15	until learning	0	Delay
25267641	150-300	15	until learning	0	Delay
25403455	280	30	600	0.2	Delay
25418414	250	12	2400	0.1	Delay
26504227	300	15	until learning	0	Delay
26551541	220	11	until learning	0.25	Delay
26655909	250	10	1000	0	Delay
26844836	250	NOT REPORTED	until learning	0	Delay
26949968	350	30	600	0.2	Delay
28533379	150-450	15	until learning	0.1	Delay
30283004	150-200	15	until learning	0	Delay
30306129	236	10	1000	0	Delay
31089195	252	NOT REPORTED	1200	0.1	Delay
31905212	250	10	2400	0.16	Delay
32015022	250	45	180	0	Delay
32294428	250	10	1200	0.1	Delay
32973240	150-450	15	480-720	0	Delay
33326350	250	10	1000	0.1	Delay
33630615	600	30	200	0.2	Delay
35589396	200	10	1400	0.2	Delay
36352508	250	NOT REPORTED	2000	0.2	Delay
36405788	150-450	12	until learning	0	Delay
36405788	150-300	NOT REPORTED	until learning	0	Delay
36405788	200	15	until learning	0	Delay and Trace
37029391	350	30	600	0	Delay
38165577	275	27.5	until learning	0.1	Delay
38565684	300	12.5	800	0.1	Delay
39150780	220	15	2000	0.08	Delay
41075059	250	20	900	0.1	Delay
41946969	200-500	4	until learning	0	Trace
42146853	400	NOT REPORTED	1000	0.1	Delay
